# MicroRNA-222 Transferred From Semen Extracellular Vesicles Inhibits Sperm Apoptosis by Targeting *BCL2L11*

**DOI:** 10.3389/fcell.2021.736864

**Published:** 2021-11-08

**Authors:** Yaqun Ding, Ning Ding, Yu Zhang, Shenmin Xie, Mengna Huang, Xiangdong Ding, Wuzi Dong, Qin Zhang, Li Jiang

**Affiliations:** ^1^National Engineering Laboratory for Animal Breeding, Key Laboratory of Animal Genetics, Breeding and Reproduction, Ministry of Agriculture, College of Animal Science and Technology, China Agricultural University, Beijing, China; ^2^College of Animal Science and Technology, Northwest A&F University, Yangling, China; ^3^College of Animal Science and Technology, Shandong Agricultural University, Tai’an, China

**Keywords:** extracellular vesicles, miR-222, sperm motility, EGFR, BCL2L11, apoptosis

## Abstract

Seminal plasma contains a large number of extracellular vesicles (EVs). However, the roles of these EVs and their interactions with sperm are not clear. To identify the important molecules affecting sperm motility in EVs, we analyzed RNA from seminal plasma EVs of boars with different sperm motility using whole-transcriptome sequencing and proteomic analysis. In total, 7 miRNAs, 67 lncRNAs, 126 mRNAs and 76 proteins were differentially expressed between the two groups. We observed that EV-miR-222 can obviously improve sperm motility. In addition, the results suggested that miR-222 was transferred into sperm by the EVs and that miR-222 affected sperm apoptosis by inhibiting the expression of *EGFR, BCL2L11, BAX, CYCs, CASP9* and *CASP3*. The results of electron microscopy also showed that overexpression of miR-222 in EVs could reduce sperm apoptosis. The study of the whole transcriptomes and proteomes of EVs in boar semen revealed some miRNAs may play an important role in these EVs interactions with Duroc sperm, and the findings suggest that the release of miR-222 by semen EVs is an important mechanism by which sperm viability is maintained and sperm apoptosis is reduced. Our studies provide a new insight of miR-222 in EVs regulation for sperm motility and sperm apoptosis.

## Introduction

Seminal plasma extracellular vesicles (SPEVs) of mammals, which are 50–500 nm in diameter, are produced mainly by the epididymis and the prostate (Saez et al., 2003) and are characterized by a high cholesterol and sphingomyelin content and a complex protein composition (Pathan et al., 2019; Saez et al., 2003; Thimon et al., 2008). As important carriers, extracellular vesicles (EVs) contain large numbers of molecules, including nucleic acids (DNA and RNA), proteins and lipids (Pathan et al., 2019; Takahashi et al., 2017). These substances may affect the gene expression patterns of recipient cells and serve as diagnostic markers for diseases. EVs play an important role in communication between cells and their surrounding microenvironment and participate in many physiologic processes, including cell proliferation and differentiation, apoptosis, immune regulation, and signaling (Kim et al., 2017).

The seminal plasma is a mixture of fluids secreted by the sexual glands and other male sexual organs and provides a nutritive and protective medium for sperm after ejaculation (Juyena and Stelletta, 2012; Druart and de Graaf, 2018). It has been found that the seminal plasma are very important for sperm metabolism, sperm function, survival and transport (Druart and de Graaf, 2018), suggesting that there are important interactions or material exchange between seminal plasma and sperm. Increasing evidence suggested that seminal plasma components are involved in a series of sperm physiology, such as sperm viability, sperm capacitation, modulation of female immune response, gamete interaction and fusion (Rodriguez-Martinez et al., 2005, 2010, 2011; Topfer-Petersen et al., 2005; Bjorndahl and Kvist, 2010; Bromfield, 2014; Schjenken and Robertson, 2014; Rath et al., 2016). For example, the interaction between seminal plasma and sperm potentially affects the longer sperm survival in humans and boars (Maxwell et al., 1999; Barranco et al., 2016; Sakaguchi et al., 2020). Some proteins in seminal plasma can impact the sperm fertilizing ability in the female genital tract, and influence the progress of pregnancy and the development of offspring (Tremellen et al., 1998; Gardner and Lane, 2005; Hao et al., 2006; Ng et al., 2010). Many studies have shown that the seminal proteins interact with the sperm cell by binding to the sperm surface and then induce membrane remodeling (Gasset et al., 2000; Greube et al., 2001; Ramakrishnan et al., 2001; Muller et al., 2002). There is also evidence that seminal plasma contains a large amount of RNA, such as mRNA, microRNA, tsRNAs (Li et al., 2012; Chen et al., 2017; Lian et al., 2021). To better understand the potential role of seminal plasma interaction with spermatozoa, a comparative analysis of small RNA profiles in pig ejaculated sperm, epididymal sperm and seminal plasma were performed. The results of this study found that ∼75% of small RNAs were shared in seminal plasma and ejaculated sperm. However, there are still some small RNAs that are ejaculated sperm specific (Chen et al., 2017). The above studies suggested that although the function of most RNAs and proteins in seminal plasma remain enigmatic, the interaction between seminal plasma and sperm has been proved to play a role in many aspects.

It has been found that the predominant seminal mRNA is contained in seminal microvesicles and shows remarkable stability (Li et al., 2012). Some sperm RNAs seem to be obtained during post-testicular maturation through active communication between sperm and epididymal and seminal EVs released by the epididymis and the male accessory sex glands (Belleannee et al., 2013; Sullivan and Saez, 2013; Sharma et al., 2016; Jodar, 2019). A recent study showed that miRNA abundance of semen-derived EVs significantly increased from the caput epididymis (7.5%) to cauda epididymis (31%), indicating miRNAs in seminal plasma EVs has important biological function (Chen et al., 2020). In addition, it has been reported that sperm can receive SPEV cargo after ejaculation and semen quality is affected by SPEVs (Murdica et al., 2019b). Recent studies demonstrated that tsRNAs and proteins were delivered from SPEVs to spermatozoa (Murdica et al., 2019b; Chen et al., 2020). Accumulating evidence shows that SPEVs play important roles in sperm function, such as sperm motility (Stegmayr and Ronquist, 1982; Fabiani et al., 1995; Arienti et al., 1999; Du et al., 2016; Murdica et al., 2019b), sperm capacitation (Pons-Rejraji et al., 2011) and the acrosome reaction (Piehl et al., 2013; Murdica et al., 2019b). Moreover, the non-coding RNAs (ncRNAs) of SPEVs have been found to be involved in the protection of sperm against the female immune response triggered in the female genital tract after contact with semen (Aalberts et al., 2014) and to be important participants in sperm-egg binding (Franchi et al., 2020). Small ncRNAs (sncRNAs) encapsulated in SPEVs can be delivered to acceptor cells from the female reproductive tract, modulating their gene expression (Robertson and Sharkey, 2016; Bai et al., 2018). Therefore, SPEVs transcriptional profiles might provide important information for the interaction between seminal plasma and sperm.

In recent years, increasing research has been performed on the microRNAs (miRNAs) and proteins in human semen EVs via RNA and proteome sequencing, and researchers have found that SPEVs are enriched with bioactive molecules, including proteins and sncRNAs such as miRNAs, Piwi-interacting RNAs (piRNAs), Y RNAs, ribosomal RNAs (rRNAs) and transfer RNAs (tRNAs) (Vojtech et al., 2014). Some miRNAs and proteins have been identified as potential biomarkers of severe diseases (Barcelo et al., 2018; Murdica et al., 2019a; Kaddour et al., 2020). For example, miR-31-5p in semen exosomes has been reported to be a highly sensitive biomarker (> 90% sensitivity) for the origin of azoospermia (Barcelo et al., 2018). MiR-196a-5p and miR-501-3p have been confirmed to be significantly downregulated in exosomes from prostate cancer (PCa) patients (Rodriguez et al., 2017). Abu-Halima et al. (2016) identified 36 differentially expressed miRNAs (DEMs) in SPEVs of 12 normal men and 12 patients with oligospermia through small-RNA sequencing (Abu-Halima et al., 2016). A total of 89 differentially expressed proteins (DEPs) were detected in exosomes of seminal plasma between normal and severely asthenozoospermic men (Murdica et al., 2019a). A recent proteomic profiling study on seminal plasma in unilateral varicocele patients showed upregulation of the annexin A2 (ANXA2) protein and downregulation of kinesin-1 h/eavy chain (KIF5B) and proposed ANXA2 and KIF5B as potential biomarkers of infertility (Panner et al., 2021).

MicroRNAs are endogenous single-stranded sncRNAs with lengths of 18-25 bases that negatively regulate gene expression at the posttranscriptional level (Kalogianni et al., 2018). MiRNAs participate in the regulation of many genes involved in reproductive processes, including reproductive tract development and function, germ cell development and maturation, fertilization and early embryo development. The unique profiles of miRNAs in exosomes in human semen show that exosomal miRNAs very likely regulate a number of reproduction-related activities (Vojtech et al., 2014).

In vertebrates, miR-222, which is encoded by a gene on the X chromosome, is highly conserved (Galardi et al., 2007). It has been reported that miR-222 plays important roles in many essential biological processes. Abnormal expression of miR-222 is closely related to the occurrence and development of various types of cancer, including lung cancer, breast cancer, liver cancer, cervical cancer, glioma and multiple myeloma (Sun et al., 2011). Moreover, miR-222 has been found to be associated with the cell cycle and apoptosis (Zhang et al., 2010). MiR-222 is also involved in the regulation of the reproductive system in male mammals. Yang et al. (2013) found that miR-221 and 222 can regulate spermatogenesis through repression of KIT expression in male mammals.

Recent studies have isolated EVs from the seminal plasma of male rats (Fornes et al., 1991), male rabbits (Davis, 1978), rams (Breitbart and Rubinstein, 1982), bulls (Frenette and Sullivan, 2001), stallions (Arienti et al., 1998), and boars (Piehl et al., 2006). Du et al. (2016) found that SPEVs of boars can be combined with sperm *in vitro* and that they promote sperm movement and prolong the effective survival time of sperm. However, the whole transcriptomes and proteomes of porcine SPEVs have not been reported, and the molecular mechanism by which SPEVs maintain sperm motility is unclear.

In the current study, we investigated the transcript and protein profiles of SPEVs from boars with different sperm motilities and identified DEMs, differentially expressed long ncRNAs (lncRNAs) (DELs), differentially expressed mRNAs (differentially expressed genes, DEGs) and DEPs between two different groups, to provide comprehensive biological information of SPEVs. In addition, we investigated the mechanism by which miR-222 is involved in the regulation of sperm motility. It is hoped that the results of this study will provide insight into the potential function of EV-microRNAs in boar semen and help in identifying important biological molecules affecting sperm motility in SPEVs.

## Materials and Methods

### Semen Collection

The sperm motility of 200 boars from a national boar facility were measured using a computer-assisted sperm analysis (CASA) system (IVOS II, France). We evaluated the sperm motility phenotypic data of three consecutive semen samples (tested once a week) for each boar. According to their semen trait records, eight Duroc boars were selected for EV extraction. All the individuals were sexually mature and were between 14 months and 22 months old. One ejaculate sample for each individual was collected by the gloved hand method. Specialized professionals obtained the sperm-rich fraction of the ejaculate from each individual, and these samples were assessed for sperm motility using the CASA system (IVOS II, France). The eight boars were divided into two groups according to their sperm motilities: the high-sperm motility group (H group) and the low-sperm motility group (L group). The boars in the H group had higher total motility (> 96%) than the boars in the L group (< 79%). Moreover, the percentages of fast-moving sperm in the H and L groups were > 72% and < 50%, respectively. Detailed information about the eight boars is shown in Supplementary Table 1. Each semen sample was centrifuged (at 800 × *g* for 15 min at 17°C) to separate sperm and supernatant. The supernatant was used to extract EVs, and the sperm were used for a coincubation experiment.

### Analysis of Sperm Motility

The semen samples collected from the eight boars were immediately assessed for sperm motility on a CASA instrument (IVOS II) in five different microscopic fields. In the coincubation experiment, sperm motility was determined visually using the CASA instrument (IVOS II) every day for seven days. After 5 min of incubation at 37°C, 7 μL of sperm suspension was placed on a prewarmed glass slide and covered with a glass cover slip. The glass slides were examined with a bright field under an optical microscope at a total magnification of 200 ×. The percentage of motile sperm was estimated in five different fields for each sample. Three separate replicates were evaluated for each semen sample, and the average of the three measurements was calculated as the final sperm motility.

### Isolation of Seminal Plasma Extracellular Vesicles

Boar SPEVs were isolated by ultracentrifugation as previously described (Piehl et al., 2013; Du et al., 2016). For each boar, 35 mL of semen plasma was centrifuged at 10000 × *g* for 30 min at 4°C to remove cellular debris and smaller pieces of undissolved seminal gel. Then, the supernatant was transferred to a clean centrifuge tube and centrifuged at 12000 × *g* for 1 h at 4°C. The supernatant was then transferred to an ultracentrifuge tube (Beckman, United States) and centrifuged at 120000 × *g* for 1.5 h at 4°C. The sediments were resuspended in DPBS (Gibco, United States), and the previous steps were repeated. Finally, the sediments were resuspended in 2 mL of DPBS, and the suspensions were filtered using 0.22 μm filters (Millipore, United States). A full description of the methodologies has been submitted to EV-TRACK (Van Deun et al., 2017).

### Transmission Electron Microscopy

A total of 20 μL of SPEV suspension was placed on a formvar carbon-coated grid for 5 min at room temperature. The grids were washed with distilled water 3 times, stained with 1.0% uranyl formate (Electron Microscope China, China) for 5 min and dried for 2 min under incandescent light. The grids were observed and photographed under a transmission electron microscope (HT770, Tokyo, Japan).

### Nanoparticle Tracking Analysis

The SPEVs were diluted to a concentration of 1 × 10^6^ ∼1 × 10^9^ particles/mL with PBS. A ZetaView PMX 110 (Particle Metrix, Meerbusch, Germany) equipped with a 405 nm laser was used to examine the sizes and quantities of particles isolated. A 1-min video shot at a frame rate of 30 frames per second was used to analyze particle motion using Nanoparticle Tracking Analysis (NTA) software (ZetaView 8.02.28).

### Western Blotting

Seminal Plasma Extracellular Vesicles were lysed with RIPA buffer (Solarbio, Beijing, China) containing 1% protease inhibitor, and the proteins were quantified with a BCA assay kit (Beyotime, China). The protein samples (15 μg) were separated by SDS-PAGE and then transferred to PVDF membranes (Millipore, United States). Then, the membranes were blocked with 5% (w/v) skim milk for 2 h; washed 5 times with TBST; incubated with antibodies primary anti-CD63 (sc-5275, Santa Cruz, CA, United States), anti-Alix (sc-53, 540, Santa Cruz, CA, United States), anti-Calnexin (10,427–2-AP, Promega, Madison, WI, United States) and anti-TSG101 (sc-13, 611, Santa Cruz, CA, United States). The membranes were incubated with secondary antibody, Proteintech product, and detected with an enhanced chemiluminescence (ECL) system.

The protein of sperms were lysed with RIPA buffer (Solarbio, China) containing 1% protease inhibitor and Membrane and Cytosol Protein Extraction Kit (Beyotime, China), and quantified with a BCA assay kit (Beyotime, China). The protein samples (30 μg) were separated by SDS–PAGE and transferred to PVDF membranes (Millipore, United States). The membranes were blocked with 5% (w/v) skim milk for 2 h, washed five times with 1 × TBST and incubated with antibodies against EGFR (18986-1-AP, Proteintech, United States), Bim (ab32158, Abcam, United Kingdom), Caspase9 (10380-1-AP, Proteintech, United States), Caspase3 (ab13847, Abcam, United Kingdom) and Tubblin (11224-1-AP, Proteintech, United States), respectively. Then, the membranes were incubated with secondary antibodies (Proteintech product), and detected with an enhanced chemiluminescence (ECL) system.

### RNA Extraction

Total RNA was extracted from SPEVs and purified using a miRNAeasy Mini Kit (Qiagen, Germany) according to a standard protocol. The RNA concentration, purity and integrity were detected using the RNA Nano 6000 Assay Kit of an Agilent Bioanalyzer 2100 System (Agilent Technologies, CA, United States).

Sperm RNA was extracted using a TRIzol Kit (Invitrogen, United States) according to the standard protocol. TRIzol (1 mL) was added to sperm and allowed to lyse the sperm for 15 min at room temperature. The samples were then centrifuged for 5 min at 12000 rpm and 4°C. Then, 200 μL of chloroform was added to the supernatants, and the samples were centrifuged at 4°C for 15 min at 12000 rpm. After discarding the precipitate, the top layer of clear liquid was added to an equal volume of isopropanol and centrifuged at 12000 rpm and 4°C for 10 min. The precipitate was washed with 1 mL of 75% alcohol and 20 μL of acryl carrier and then centrifuged at 7500 rpm and 4°C for 5 min. Finally, ddH_2_O without RNase was added to dissolve the precipitate prior to storage at −80°C.

### Whole-Transcriptome Sequencing

Deep RNA sequencing was performed to gain insight into the types of RNA in SPEVs, including mRNAs, miRNAs and lncRNAs. Small-RNA and long-RNA libraries were established. For the long-RNA sequencing libraries, a total of 250 pg to 10 ng of RNA per sample was used as input material using a SMARTer Stranded Total RNA-Seq Kit (Takara Bio Inc., United States) following the manufacturer’s recommendations, and index codes were added to attribute the sequences to each sample. For the small-RNA sequencing libraries, a total of 1 ng to 500 ng of RNA per sample was used as input material. The sequencing libraries were generated using a QIAseq miRNA Library Kit (Qiagen, Frederick, MD, United States) according to the manufacturer’s instructions, and index codes were added to attribute the sequences to each sample. Library quality was assessed on an Agilent Bioanalyzer 2100 and by qPCR. Clustering of the index-coded samples was performed on an acBot Cluster Generation System using a TruSeq PE Cluster Kitv3-cBot-HS (Illumina, San Diego, CA, United States) following the manufacturer’s protocol. Finally, the library preparations were sequenced on an Illumina HiSeq platform, and paired-end reads were generated.

### Profiling of mRNA and Long ncRNAs in Seminal Plasma Extracellular Vesicles

The clean data were aligned to the Sscrofa11.1 reference genome with HISAT2 (Kim et al., 2015). Transcripts from all samples were then assembled with StringTie (Pertea et al., 2015). The lncRNAs were identified. Briefly, transcripts whose class codes were “i,” “x,” “u,” “o,” and “e” were retained. Transcripts with lengths greater than 200 nt and more than two exons were selected as lncRNA candidates and further screened (Kelley and Rinn, 2012). Four computational resources, including the CPC (Kong et al., 2007), CNCI (Sun et al., 2013), Pfam (Finn et al., 2014) and CPAT (Wang et al., 2013), were used together to sort non-protein coding RNA candidates from lncRNA transcripts (with fragments per kilobase million (FPKM) values ≥ 0.1) obtained in the previous step. We used StringTie (Pertea et al., 2015) to analyze the expression levels of lncRNAs and mRNAs and edgeR (Robinson et al., 2010) to identify DELs and DEGs with the criteria of a | Fold change (FC)| ≥ 1.5 and *P* ≤ 0.05. We used two methods to predict the target genes of the DELs. First, according to the predicted positions of lncRNAs and genes, we selected the neighboring genes within 100 kb of the DELs as the target genes; second, according to the complementary base pairing of lncRNAs and mRNAs, we used the target gene prediction tool of LncTar (Li et al., 2015) to predict the target genes of our DELs.

### Profiling of MicroRNAs in Seminal Plasma Extracellular Vesicles

We used Bowtie (Langmead and Salzberg, 2012) software to align the clean reads to the Silva (Quast et al., 2013), GtRNAdb (Chan and Lowe, 2016), Rfam (Kalvari et al., 2018) and Repbase (Jurka, 2000) databases in order to filter the rRNAs, tRNAs, small nuclear RNAs (snRNAs), small nucleolar RNAs (snoRNAs) and other ncRNAs and repeats. Sscrofa11.1 was used as the reference genome^1^. We compared the read sequences of the reference genome with the mature miRNA sequences in miRBase (V22) (Kozomara and Griffiths-Jones, 2014) to identify known miRNAs and predicted the new miRNAs using miRDeep2 (Friedlander et al., 2012) software. We used edgeR software to analyze the DEMs with | FC| -values ≥ 1.5 and *P*-values ≤ 0.05. According to the known miRNAs, the newly predicted miRNAs and the gene sequence information of the corresponding species, we used MiRanda and RNAhybrid to predict the target genes of the DEMs.

### LC-MS/MS Analysis and Data Processing

Seminal Plasma Extracellular Vesicle samples were lysed with 8 M urea and 100 mM Tris–HCl (pH 8) buffer, and the proteins were quantified by BCA assay (Osti et al., 2019). The SPEV protein samples (∼60 μg per sample) were digested with trypsin. The digested peptide mixtures were resuspended in buffer A (0.1% formic acid, FA) and then separated on an UltiMate 3000 HPLC System (Thermo Fisher Scientific, Waltham, MA, United States) coupled online to an Orbitrap Fusion Lumos mass spectrometer (Thermo Fisher Scientific, Waltham, MA, United States). The peptides were subjected to a C18 trap column (3 μm, 100 μm × 20 mm) at a flow rate of 0.6 μL/min, desalted online and loaded into a C18 column (1.9 μm, 150 μm × 120 mm) for 75 min using a gradient of 7–100% buffer B (0.08% FA and 80% acetonitrile, ACN). The mass spectrometer was operated in positive mode using a data-dependent acquisition method. A full MS scan (300–1400 m/z) was acquired in the Orbitrap with the resolution set to a value of 120000.

The raw spectra were searched against the Proteome Discover database. Trypsin was selected as the proteolytic enzyme, and two missed cleavages were allowed. Cysteine carbamidomethyl was set as the fixed modification, and methionine oxidation and N-terminal acetylation were set as the variable modifications. A peptide mass tolerance of 10 ppm and a fragment mass tolerance of 0.6 Da were used for search engines. The false discovery rates (FDRs) of the peptide-spectrum matches (PSMs) and proteins were set to less than 1%. Data analysis was performed in the R software environment. An | FC| ≥ 1.5 and a *P* ≤ 0.05 were set as the thresholds for significant DEPs between the H group and the L group. In addition, proteins identified in only one group were considered specifically expressed proteins, and they were also deemed DEPs.

### Gene Ontology and Kyoto Encyclopedia of Genes and Genomes Pathway Enrichment Analysis

For all the DEMs between the H group and the L group, the potential target genes were predicted with both RNAhybrid and miRanda. The target genes found with both software packages were subjected to Gene Ontology (GO) and Kyoto Encyclopedia of Genes and Genomes (KEGG) pathway analysis. For each DEL between the two groups, the potential target genes were predicted with LncTar software and Perl scripts. GO enrichment and KEGG pathway analysis were performed for the target genes of the DEMs, DELs and for the DEGs and DEPs, respectively. The GO enrichment and KEGG pathway enrichment were analyzed using KOBAS software (Xie et al., 2011).

### Quantification of MicroRNA, mRNA and Long ncRNA Expression With qPCR

Total RNA (5 ng) from each sample was used for miRNA reverse transcription using an ABI TaqMan^TM^ MicroRNA Reverse Transcription Kit (Thermo Fisher Scientific Inc., United States) according to the manufacturer’s instructions. A total of 500 ng of total RNA from each sample was used for mRNA reverse transcription using a Takara Prime-Script^TM^ RT Reagent Kit with gDNA Eraser (Takara Bio Inc., Japan) following the manufacturer’s protocols. qPCR was performed using a LightCycler 480 SYBR Green I Master kit on a Roche LightCycler 480 instrument for miRNA, mRNA and lncRNA quantification. All qPCR amplifications were carried out in a 96-well plate with a final reaction volume of 20 μL containing 1 μL of forward and reverse primers (10 pM/μL), 2 μL of cDNA (300 ng/μL), 10 μL of Master Mix (2 ×) and 6 μL of ddH_2_O. qPCR was carried out on each sample in triplicate. The miRNAs were standardized with U6, and the mRNAs and lncRNAs were standardized with glyceraldehyde phosphate dehydrogenase (*GAPDH*) and actin beta (*ACTB*). All primers used for real time qPCR was shown in the Supplementary Table 2.

### Coincubation Experiment

To investigate whether SPEVs from the H group and L group had different effects on sperm motility, we incubated sperm with SPEVs at 17°C for seven days. Semen samples were centrifuged (at 800 × *g* for 10 min at 17°C) to separate sperm. Then, the sperm pellets were resuspended, and the sperm concentration was adjusted with Modena diluent to 1 × 10^8^ sperm/mL. The Modena diluent was composed of 2.75 g of glucose, 0.235 g of EDTA-Na_2_, 0.69 g of sodium citrate, 0.29 g of citric acid, 0.1 g of NaHCO_3_, and 0.665 g of Tris per 100 mL of ultrapure water (pH = 7.2) (Estienne et al., 2007). EVs were extracted from the seminal plasma of boars, and the concentrations of SPEVs were detected with a ZetaView PMX 110 (Particle Metrix, Meerbusch, Germany). The control group samples were diluted with a diluent lacking EVs, and the samples in the H and L groups were diluted with a diluent containing 2 × EVs (2 times the average EV concentration of the original semen). Finally, the semen samples were stored in an incubator at 17°C in an atmosphere of 5% CO_2_ and saturated humidity.

To investigate the function of miR-222 in SPEVs, we also coincubated sperm cells with electrotransfected SPEVs at 17°C. This experiment included three groups: a miR-222 mimic group, a miR-222 inhibitor group and a group of SPEVs without electrotransfection (the control group). Sperm samples were collected on day 1 and day 4.

### Seminal Plasma Extracellular Vesicle Electrotransfection

To investigate the function of miR-222 in SPEVs, SPEVs were electroporated with a miR-222 mimic or miR-222 inhibitor purchased from Shanghai Gene Pharma (China). The SPEVs were electroporated with 400 pmol of the miR-222 mimic (sense: 5′-AGCUACAUCUGGCUACUCGGGUCC-3′; antisense: 5′-GACCCAGUAGCCAGAUGUAGCUUU-3′) or 800 pmol of the miR-222 inhibitor (sense: 5′-GAGACCCAGUAGCCAGAUGUAGCU-3′) using a Gene Pulser Xcell electroporator (Bio-Rad, United States) with the “exponential” protocol (250 V, 100 μF, R = ∞, cuvette size = 4 mm) (Vu et al., 2019). The electroporated SPEVs were mixed with the diluent and added to sperm. The control-group sperm were diluted with a diluent containing 2 × SPEVs without electroporation, and the sperm in the other two groups were diluted with a diluent containing 2 × SPEVs electroporated with the miR-222 mimic or inhibitor. All the samples were incubated at 17°C in an atmosphere of 5% CO_2_ and saturated humidity. To evaluate the efficiency of electroporation, RNA was extracted from SPEVs without electroporation or SPEVs electroporated with the miR-222 mimic or miR-222 inhibitor, and the miR-222 levels were measured using real-time qPCR.

### Ultrathin Sectioning and Electron Microscopy

To further observe the effect of miR-222 on sperm, ultrathin sections of sperm were made after coincubation with SPEVs. One group was coincubated with SPEVs transfected with the miR-222 mimic, and the other group was coincubated with SPEVs transfected with the miR-222 inhibitor.

The sperm samples were centrifuged at 17°C and 800 × *g* for 5 min, and the supernatant was discarded. Then, the samples were placed in a fixative solution of pH = 7.2 [a mixture of 2% PFA (Alfa Aesar, United States) and 2.5% glutaraldehyde (SPT Supply, United States)] and placed on ice for approximately 1 h. Then, the samples were fixed in 1% osmic acid on ice for 1 h. Next, 1% uranyl acetate (Electron Microscope China, China) was used for staining. The samples were washed with PBS three times after each step. The samples were then dehydrated with different concentrations of alcohol and embedded in epoxy resin (Electron Microscopy Sciences, United States). Finally, an ultrathin slicer (EMUC7, Leica, Germany) was used to slice the blocks at a thickness of 70 nm. After cutting, the sections were imaged by electron microscopy (H-7650B, Tokyo, Japan).

### Luciferase Reporter Assay

We used RNAhybrid and miRanda to predict miR-222 target genes and chose *NASP*, *LHX8*, *BCL2L11* and *EGFR* for a luciferase reporter assay. The wild-type gene fragments of the 3’UTR region were amplified by PCR with an upstream primer within the *Sac*I site and a reverse primer within the *Xba*I site using PrimeSTAR polymerase (Takara, China, Japan), and purified with a TIANgel Midi Purification Kit (Tiangen, China). The products were digested by the restriction endonucleases *Sac*I and *Xba*I (TransGen, China), and the inserts were linked to the pmirGLO double luciferase reporter vector with a DNA ligation kit (Takara, China). The constructs were validated by sequencing. Mutant vectors were created from the wild-type vectors by using a Fast Mutagenesis System (TransGen, China). We designed mutation primers (Supplementary Table 3) to change the seed sequences (the miRNA-gene binding sites). The resulting reporter vectors are termed NASP-WT, LHX8-WT, BCL2L11-WT and BCL2L11-MUT, EGFR-WT and EGFR-MUT.

The constructed plasmids were cotransfected with the miR-222 mimic or inhibitor into 293T cells using Lipofectamine 2000 (Invitrogen, United States). Forty-eight hours after transfection, luciferase activity was detected by an enzyme labeling instrument (Tecan, Switzerland) according to the specifications of the Dual Luciferase Reporter Assay System (Promega, United States).

### Cell Culture and Cell Transfection

293T cells were incubated in DMEM/F12 (Gibco, United States) supplemented with 5% fetal bovine serum (FBS, BI, Israel) and 100 U/mL penicillin-streptomycin (Solarbio, China) at 37°C in a humidified 5% CO_2_ atmosphere. Two days later, when the cell density had reached 90%, the cells were passaged. The 293T cells were detached from the culture plates with 0.25% trypsin (Gibco, United States) and transferred to 24-well plates with antibiotic-free medium. The transfection solution was prepared by mixing 2 μL of Lipofectamine 2000 (Invitrogen, Carlsbad, CA, United States), 12.5 nM miR-222 mimic or 25 nM miR-222 inhibitor, and 0.5 μg of vector and adding MEM supplement to a volume of 100 μL. After 4 h of transfection, the transfection solution was discarded and replaced with fresh medium. After 48 h of transfection, the cells were collected for detection.

### Statistical Analysis

Statistical tests were performed using the Statistical Package for the Social Sciences (SPSS) for Windows, release 21.0 (SPSS Inc., Chicago, IL, United States). Two groups were compared by *t*-test, and multiple groups were compared by ANOVA. The FDR was controlled for multiple comparisons. The data are presented as the mean ± standard error. *P* ≤ 0.05, *P* ≤ 0.01 and *P* ≤ 0.001 based on at least 3 replicates were considered to indicate statistical significance.

## Results

### Isolation and Characterization of Boar Seminal Plasma Extracellular Vesicles

**FIGURE 1:**
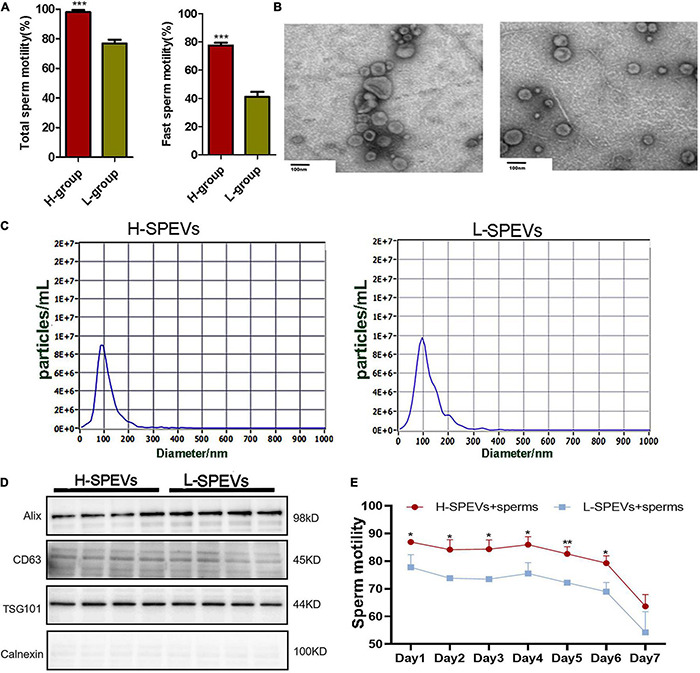
Isolation and characterization of SPEVs from boars. **(A)** The difference of total sperm motility and fast motility between the two groups. **(B)** TEM images of SPEVs. Scale bars: 100 nm. **(C)** NTA results showing that the EVs enriched from seminal plasma were approximately 50–200 nm in diameter. **(D)** EV markers Alix, Tsg101 and CD63 were all detected in L-SPEVs and H-SPEVs. Calnexin, a negative marker of EVs, was not present in our isolated SPEV samples. **(E)** Line chart comparing the motility of Duroc boar sperm incubated with H-SPEVs and L-SPEVs. Data are presented as mean ± SEM. **(A)** ***P ≤ 0.001 (Student’s *t*-test), *n* = 4 per group. **(E)** *P ≤ 0.05, **P ≤ 0.01, ***P ≤ 0.001 (Student’s *t*-test), *n* = 3 per group.

Phenotypic analysis showed that there were significant differences in the total sperm motility and fast sperm motility between the two groups (Figure 1A). SPEVs were isolated from boars with high or low sperm motility by the ultracentrifugation method. Most SPEVs appeared intact and were typically cup-shaped (Figure 1B). To characterize the properties of SPEVs, NTA was used to assess the size distribution of SPEVs. The mean size of the SPEVs was 108.7 nm, and the particle size ranged from 50 nm to 200 nm (Figure 1C). Western blot analysis showed that Alix, CD63 and TSG101 (EV markers) were all detected in SPEVs isolated from four boars in the H group and four boars in the L group. In contrast, Calnexin, a negative marker of EVs, was not present in the SPEVs of eight boars (Figure 1D).

### The Seminal Plasma Extracellular Vesicles of the High-Sperm Motility Group Improved Sperm Motility

To determine whether the SPEVs of the H and L groups had different effects on sperm motility, we added SPEVs from the H group and L group (H-SPEVs and L-SPEVs, respectively) to sperms from Duroc boars with high sperm motility (*n* = 3). The concentrations of SPEVs in the H-SPEV and L-SPEV groups were adjusted to 2-time the EV concentrations of the original seminal plasma. Then, the effects of the different SPEVs on sperm motility were measured for seven days. The results showed that the motility of sperm incubated with H-SPEVs was significantly higher than that of sperm incubated with L-SPEVs (Figure 1E).

### Comparison of the Whole Transcriptomes of Seminal Plasma Extracellular Vesicles Between the High-Sperm Motility and Low-Sperm Motility Groups

To explore the mechanism of the effect of EVs on sperm motility, we analyzed the whole transcriptomes of SPEVs from the two groups with different sperm motilities using next-generation sequencing. Small-RNA, mRNA and lncRNA libraries were constructed and sequenced. A total of 178.51 million raw reads were obtained for small RNAs, and the percentage of mapped reads for each sample was approximately 80% (Supplementary Table 4). In addition, 203.44 Gb of clean data were obtained for mRNAs and lncRNAs. The mapped reads accounted for 82.21–88.89% of the long-RNA libraries (Supplementary Table 5).

**FIGURE 2:**
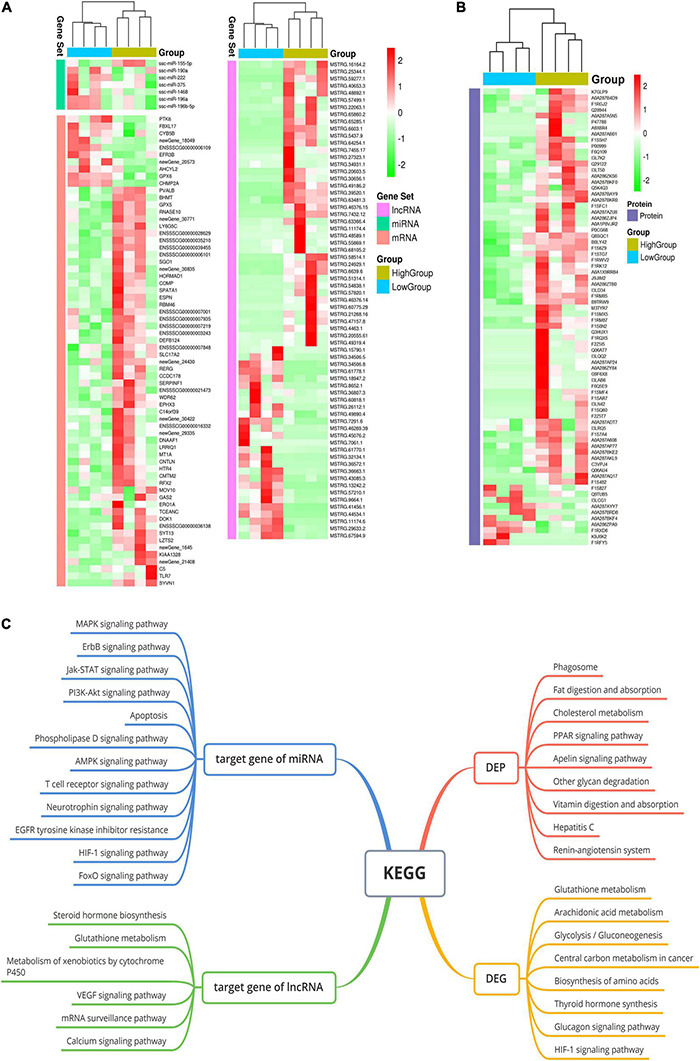
The whole-transcriptome profiles of the H-SPEV and L-SPEV groups. **(A)** Heatmap of the 7 DEMs, 126 DEGs and 67 DELs in SPEVs between the two groups. **(B)** Heatmap of 76 DEPs in SPEVs between the two groups. **(C)** GO and KEGG enrichment of DEGs, DEPs, DEM target genes and DEL target genes. The significant GO terms and KEGG pathways are shown in the graph.

The reads obtained from the small-RNA libraries were mapped to an annotated miRNA database (miRBase), and many different types of small RNAs, such as miRNAs, piRNAs, rRNAs, scRNAs, snoRNAs, snRNAs and tRNAs, were detected in boar SPEVs (Supplementary Figure 1). Approximately 50% of the mapped reads represented miRNAs. In total, 528 miRNAs were detected in all the samples. The miRNAs in all samples were mainly 21–23 nt in length (Supplementary Figure 2). In total, 345 out of 528 miRNAs were confirmed with the miRbase database^2^, and 183 miRNAs were determined to be novel. Seven DEMs (miR-222, miR-155-5p, miR-190a, miR-1468-5p, miR-196a-5p, miR-196b-5p and miR-375) were detected between the H group and the L group (| FC| ≥ 1.5, *P* ≤ 0.05) (Figure 2A and Supplementary Figure 3).

With regard to mRNAs, we found 126 DEGs between the H group and the L group (Figure 2A and Supplementary Table 6). Most of the DEGs (105 out of 126) showed higher expression in the H group than in the L group (| FC| ≥ 1.5, *P* ≤ 0.05). With regard to lncRNAs, a total of 23144 lncRNAs were identified in all the samples, including 59.6% lincRNAs, 19.7% antisense lncRNAs, 13% intronic lncRNAs and 7.7% sense lncRNAs (Supplementary Figure 4). Sixty-seven lncRNAs were significantly differentially expressed between the two groups (| FC| ≥ 1.5, *P* ≤ 0.05) (Figure 2A and Supplementary Table 7).

To gain further insight into the pathways represented by the 126 DEGs and the target genes of the DEMs and DELs, we performed GO enrichment analysis and pathway analysis using KOBAS software. The analyses of the DEGs between the H group and the L group revealed 20 significant GO terms and eight significant pathways (*P* ≤ 0.05) (Figure 2C). The two most significant GO terms were the sperm motility and flagellated sperm motility terms. Other GO terms were associated with cilium-dependent cell motility, mitochondrial gene expression, carbohydrate metabolic processes, hexose metabolic processes, extracellular transport, oxidation-reduction processes and NADPH regeneration. Pathway analysis revealed that the three most significant pathways were the glutathione metabolism, arachidonic acid metabolism and glycolysis/gluconeogenesis pathways. With regard to the target genes of the DEMs between the H group and the L group, pathway analysis revealed 77 significant pathways (corrected *P* ≤ 0.05) (Figure 2C). These pathways were mostly related to the Jak-STAT signaling pathway, the PI3K-Akt signaling pathway, the MAPK signaling pathway, the ErbB signaling pathway, apoptosis, the FoxO signaling pathway, focal adhesion and PCa. The target genes of the DELs were enriched in eleven pathways (*P* < 0.05), such as the steroid hormone biosynthesis, glutathione metabolism, VEGF signaling and calcium signaling pathways (Figure 2C).

### Proteomic Profiles of Seminal Plasma Extracellular Vesicles Derived From the High-Sperm Motility and Low-Sperm Motility Groups

To further investigate the relationships between exosomal proteins and sperm motility, we performed proteomics analysis of SPEVs from the two groups with different sperm motilities. In total, 1435 exosomal proteins, including 9409 non-redundant peptides, were identified in the SPEVs of all samples with a highly conservative threshold (confidence ≥ 95%, at less 2 unique peptides matched and FDR < 1%). The total proteins covered a broad range of protein classes, such as enzymes, cytoskeletal proteins, extracellular proteins and transcription factors.

Our results showed that 76 out of the 1435 proteins were differentially expressed in SPEVs between the H and L groups (| FC| ≥ 1.5, *P* ≤ 0.05) (Figure 2B and Supplementary Table 8). A total of 38 proteins were downregulated in the L group compared with the H group, while 6 proteins were significantly upregulated. In addition, 28 proteins were identified only in the H group, and 4 proteins were identified only in the L group. The DEPs between the two groups are shown in Supplementary Table 8. The GO and KEGG analyses showed that the DEPs were associated primarily with negative regulation of cell motility, extracellular exosomes, the extracellular space, cell-cell adhesion and phagosome biological activities. Furthermore, there were significant differences in the expression levels of two genes, *UTRN* and *LY6G5C*, at both the transcriptomic and proteomic levels. In particular, the mRNA and protein expression levels of UTRN were higher in the L group than in the H group, whereas LY6G5C showed lower mRNA and protein expression levels in the boars of the L group than in those of the H group.

### Construction of Competing Endogenous RNA Regulatory Networks for Sperm Motility

**FIGURE 3:**
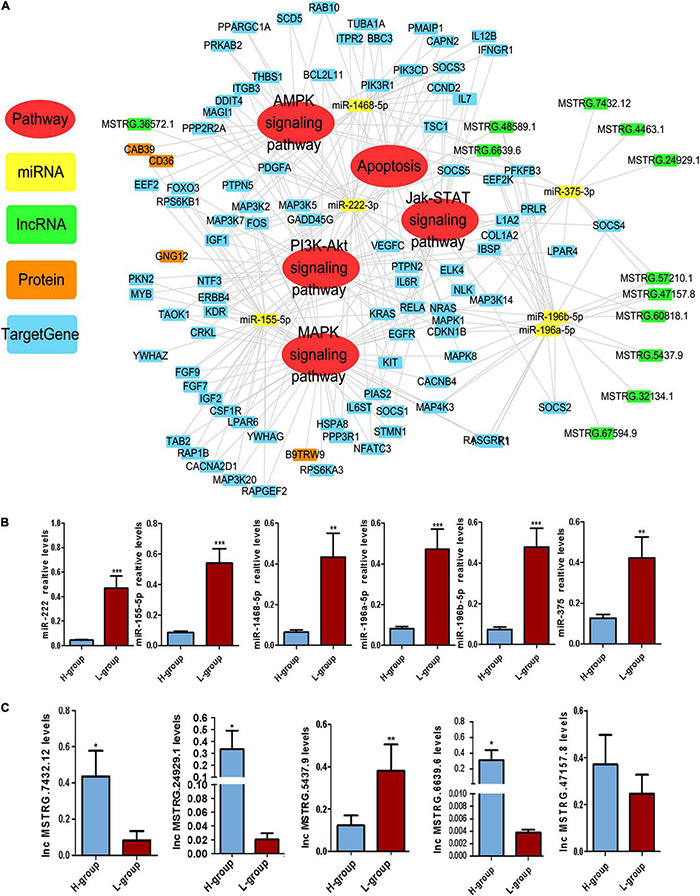
Competing Endogenous RNA (CeRNA) regulatory networks in Duroc boars with different sperm motility. **(A)** The ceRNA network was based on DEMs, DELs and DEM target genes. The network consisted of five signaling pathways, including the AMPK, apoptosis, Jak-STAT, PI3K-AKT, and MAPK signaling pathways. The DEMs, DELs, DEPs and DEM target genes in the network are presented in different colors. **(B)** qPCR validation results for DEMs in the ceRNA network. **(C)** qPCR validation results for DELs in the ceRNA network. *P ≤ 0.05, **P < 0.01, ***P ≤ 0.001 (Student’s *t*-test), *n* = 4 for each group.

Based on the GO and KEGG analyses of the target genes of the DEMs and DELs, five important pathways affecting sperm function were selected for construction of Competing endogenous RNA (ceRNA) regulatory networks (Figure 3A), including the AMPK, apoptosis, Jak-STAT, PI3K-AKT and MAPK signaling pathways. Twelve DELs specifically targeted by 5 DEMs and 23 miRNA-lncRNA interaction pairs were found. Furthermore, four DEPs were also found in these five pathways, including CAB39, CD36, GNG12 and B9TRW9.

In this ceRNA network, we selected six important DEMs and five important DELs for verification. qPCR was performed to verify the expression of these DEMs and DELs in H-SPEVs and L-SPEVs. The results showed that six miRNAs were significantly differentially expressed between the two groups (Figure 3B). In addition, the six miRNAs were all upregulated in the L-SPEV group. Moreover, the qPCR results also showed that four lncRNAs were significantly differentially expressed between H-SPEVs and L-SPEVs, and the expression trends of all lncRNAs in the two groups were consistent with the sequencing results (Figure 3C).

### MiR-222 Is Transferred to Sperm via Extracellular Vesicles

**FIGURE 4:**
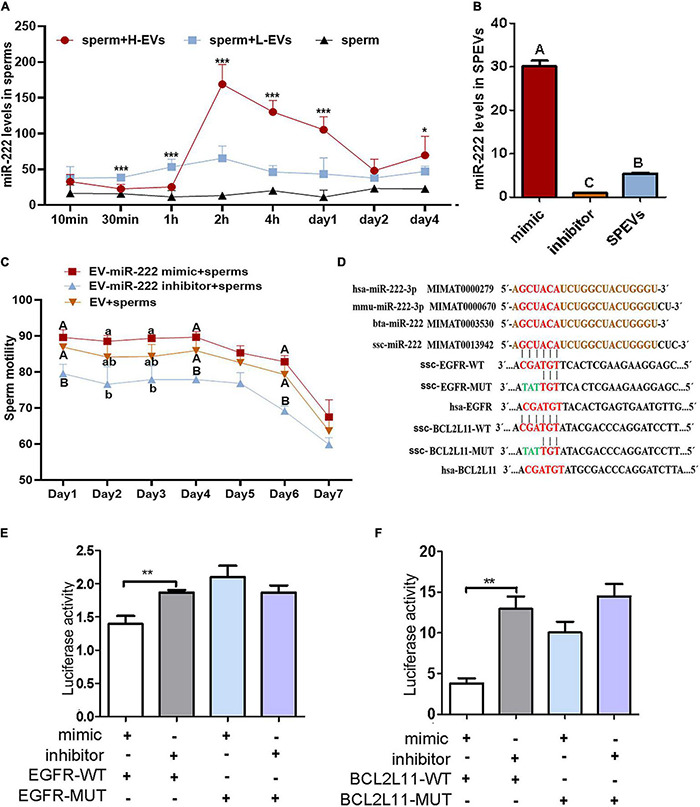
miR-222 targets *EGFR* and *BCL2L11*. **(A)** The relative miR-222 levels in sperm from healthy boars that were incubated with H-SPEVs and L-SPEVs, respectively. **(B)** The efficiency of electroporation was determined by qPCR. **(C)** The total motility of sperm incubated with EV-miR-222 mimic, EV-miR-222 inhibitor and SPEV-NC, respectively. **(D)** The sequence of miR-222 is conserved in many species. The predicted target-binding sites between miR-222 and *EGFR* and between miR-222 and *BCL2L11* are shown. **(E)** A luciferase activity assay showed that miR-222 targets the *EGFR* 3′UTR. **(F)** A luciferase activity assay showed that miR-222 targets the *BCL2L11* 3′UTR. *P ≤ 0.05, **P < 0.01, ***P ≤ 0.001 (Student’s *t*-test), different capital letters indicate significantly different values at *P* ≤ 0.01, different lowercase letters indicate significantly different values at *P* ≤ 0.05 (one-way ANOVA), *n* = 3 for each group.

Among the six DEMs, we observed that miR-222 was an important hub node in the ceRNA network and had the highest connectivity (degree = 37). The sequencing results showed that miR-222 exhibited the highest expression level among the six DEMs (Supplementary Figure 3). In addition, miR-222 is located on the X chromosome and is highly conserved in many species, indicating that it may be related to sperm function. To study the effect of EV-derived miR-222 (EV-miR-222) on sperm, sperms were incubated with SPEVs from three boars of H group and three boars of L group. Then, the expression of miR-222 in sperm on 10 min, 30 min, 1 h, 2 h, 4 h, day 1, day 2 and day 4 of incubation was detected using qPCR. Our results showed that the expression of miR-222 in sperm incubated with H-SPEVs was significantly lower than that in sperm incubated with L-SPEVs before 1 h (Figure 4A). However, beginning on the 2 h, the expression of miR-222 in sperm incubated with H-SPEVs was significantly higher than that in sperm incubated with L-SPEVs (Figure 4A). In addition, we observed that the expression level of miR-222 was the highest level at 2 h in sperms incubated with H-SPEV or L-SPEV (Figure 4A). Therefore, our results suggested that SPEVs might be taken up by sperm within 2 h. Furthermore, compared with L-SPEVs, H-SPEVs significantly and persistently increased the expression of miR-222 in sperm after 2 h.

### EV-Derived miR-222 Transferred to Sperm Improved Sperm Motility

To determine the function of miR-222 on sperm motility, SPEVs were elctroporated with the miR-222 mimic or miR-222 inhibitor. To evaluate the efficiency of electroporation, the miR-222 levels of SPEVs without electroporation and SPEVs electroporated with the miR-222 mimic or miR-222 inhibitor were measured using real-time qPCR (Figure 4B). The results suggested that miR-222 mimic or inhibitor was successfully transferred to EVs (Figure 4B). Then, we added 2-time EV-miR-222 mimic, EV-miR-222 inhibitor or EV to the sperms of the same Duroc boar, respectively (*n* = 3). Our results showed that EV-miR-222 mimic significantly increased the sperm motility compared with EV-miR-222 inhibitor (Figure 4C). In addition, there was a significant difference (*P* < 0.01) in sperm motility between EV-miR-222 mimic group and EV-miR-222 inhibitor group on the first day and the fourth day (Figure 4C). The results suggested that EV-miR-222 can improve sperm motility.

### MiR-222 Targets EGFR and BCL2L11 to Downregulate Their Expression

We next addressed the molecular mechanism by which miR-222 regulates sperm motility. The miR-222 sequence is highly conserved in a variety of species according to miRBase see text footnote 2, including pigs, humans, mice and cattle (Figure 4D). We accordingly searched for potential mRNA targets of miR-222 using RNAhybrid. The 3’UTRs of four predicted candidate genes (*EGFR*, *BCL2L11*, *NASP* and *LHX8*) were found to contain a miR-222 target-binding site (Figure 4D and Supplementary Figure 5). To directly test whether these candidates were target genes of miR-222, their 3′UTRs were cloned into luciferase reporter vectors, and the resulting constructs were cotransfected with miR-222 mimic or inhibitor into 293T cells. Luciferase expression effectively decreased upon coexpression of miR-222 with the *EGFR* 3′UTR or the *BCL2L11* 3’UTR (Figures 4E,F). To test whether the predicted miR-222 target sites in the *EGFR* 3′UTR and *BCL2L11* 3′UTR are critical for the miR-222-mediated inhibition of *EGFR* expression, we introduced mutations into the seed sequences of predicted miR-222 binding sites. These mutations within the *EGFR* 3′UTR and *BCL2L11* 3′UTR abolished the inhibitory effects of miR-222 on luciferase expression (Figures 4E,F), suggesting that *EGFR* and *BCL2L11* are directly regulated by miR-222. However, both the miR-222 mimic and inhibitor had no effect on the luciferase activity of wild-type *LHX8* and *NASP* (Supplementary Figure 5).

### EV-Derived miR-222 Transferred to Sperm Inhibits Sperm Apoptosis

**FIGURE 5:**
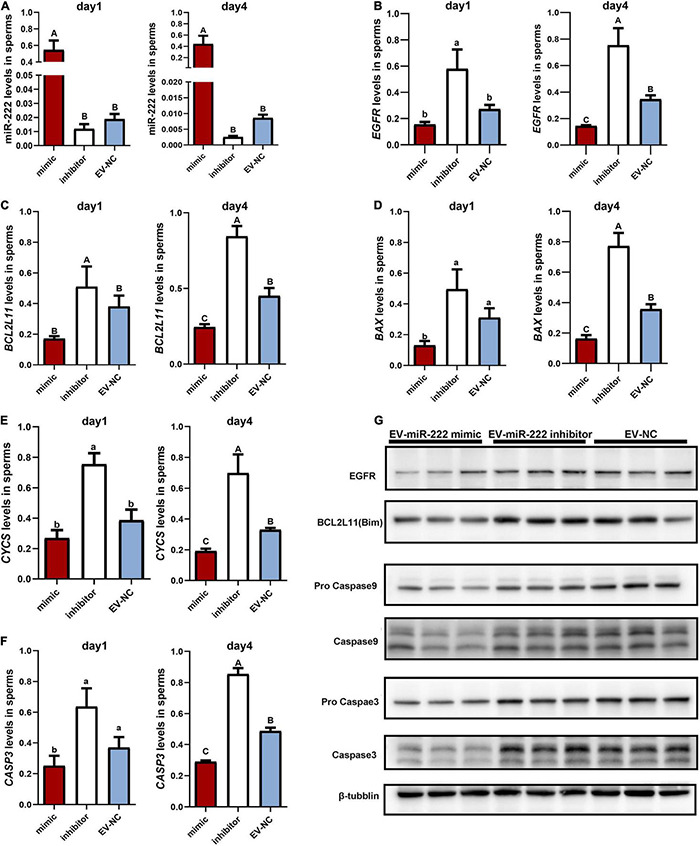
Effect of miR-222 on porcine sperm *in vitro*. **(A)** The relative expression levels of miR-222 in sperm incubated with SPEVs for one day and four days were determined by qPCR. **(B–F)** The relative expression levels of *EGFR* **(B)**, *BCL2L11* **(C)**, *BAX* **(D)**, *CYCs* **(E)** and *CASP3* **(F)** in sperm incubated with EV-miR-222 mimic, EV-miR-222 inhibitor and EV-NC for one day and four days were determined by qPCR. **(G)** The protein expression levels of EGFR, BCL2L11 (Bim), pro Caspase9 and Caspase9, pro Caspase3 and Caspase3, and tubulin in sperm incubated with EV-miR-222 mimic, EV-miR-222 inhibitor and EV-NC for four days were determined by WB. From **(A–F).** data are presented as mean ± SEM. Different capital letters indicate significantly different values at *P* ≤ 0.01, different lowercase letters indicate significantly different values at *P* ≤ 0.05 (ANOVA), *n* = 3 for each group.

According to the phenotype of sperm incubated with EV-miR-222 mimic and inhibitor, the first and fourth days with the most significant difference in sperm motility were selected for follow-up study. We detected the expression of miR-222 in sperms cultured with EV-miR-222 mimic or EV-miR-222 inhibitor for one day and four days. Our results showed that the expression level of miR-222 in sperms cultured with SPEVs electroporated with the miR-222 mimic was significantly higher than that in sperms cultured with SPEVs electroporated with the miR-222 inhibitor or that in control sperms (Figure 5A). The results suggested that miR-222 was successfully transferred to sperm through EVs.

As demonstrated above, miR-222 regulated the mRNA expression of *EGFR* and *BCL2L11*. To evaluate the role of miR-222 overexpression or inhibition in sperm, we first performed qPCR assays to detect *EGFR* and *BCL2L11* mRNA levels in sperms incubated with EV-miR-222 mimic or miR-222 inhibitor for one day and four days. The results showed that the mRNA expression levels of *EGFR* and *BCL2L11* were significantly lower in sperms incubated with SPEVs electroporated with the miR-222 mimic than in sperm incubated with SPEVs electroporated with the miR-222 inhibitor (Figures 5B,C). Furthermore, EGFR and BCL2L11 protein expression levels in sperms at four day were detected using Western Blot. The results showed that the protein expression levels of EGFR and BCL2L11 were also obviously lower in sperm incubated with EV-miR-222 mimic than in sperm incubated with EV-miR-222 inhibitor (Figure 5G). This result suggests that miR-222 inhibits the expression of EGFR and BCL2L11 in sperm. BCL2L11 is a mitochondrial member of the BCL-2 family and acts as an important regulator of apoptosis (Suhaili et al., 2017). Previous studies have shown that BCL2L11 directly activates the proapoptotic member BAX to change the permeabilization of the mitochondrial outer membrane and finally leads to the release of Cyt-C (*CYCs*) from mitochondria into the cytoplasm. Therefore, we detected the expression levels of *BAX, CYCs* and *CASP3* in different groups. We observed that the expression levels of *BAX, CYCs* and *CASP3* in sperm incubated with EV-miR-222 mimic were significantly lower than those in sperm incubated with EV-miR-222 inhibitor (Figures 5D–F). Similarly, the protein expression level of CASP9 and CASP3 was significantly decreased in sperm incubated with EV-miR-222 mimic (Figure 5G), indicating that high expression of miR-222 can inhibit sperm apoptosis.

**FIGURE 6:**
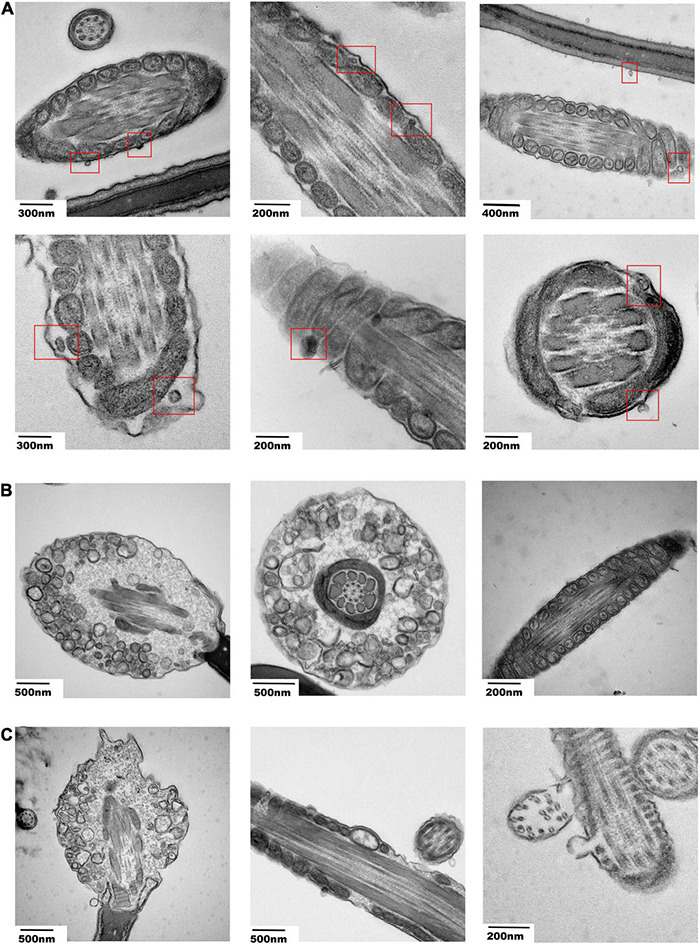
Electron microscopy images of sperm incubated with transfected SPEVs. **(A)** Electron microscopy images showing the vesicle-like structures near the mitochondria within the cell membrane of the sperm tail. **(B)** Electron microscopy images showing the sperm and the shapes of mitochondria after incubation with the EV-miR-222 mimic. **(C)** Electron microscopy images showing the sperm and the shapes of mitochondria after incubation with the EV-miR-222 inhibitor.

To observe the effect of miR-222 on sperm, sperm incubated with EV-miR-222 mimic and EV-miR-222 inhibitor for one day were cut into slices. We observed some vesicle analogs with diameters of approximately 70–100 nm around the mitochondria in the tails of sperm (Figure 6A). Moreover, Transmission Electron Microscopy (TEM) images suggested that sperm cultured with EV-miR-222 inhibitor (Figure 6C) showed obvious apoptosis, in strong contrast to those cultured with EV-miR-222 mimic (Figure 6B). Intact sperm membrane were observed in the mimic group, but the sperm membrane in the inhibitor group were significantly shrunken and broken (Figures 6B,C). In the miR-222 inhibitor group, vacuole-like mitochondria were found, and mitochondrial deletion occurred (Figure 6C). These results suggest that SPEVs transfer miR-222 into sperm and that miR-222 inhibits sperm apoptosis.

## Discussion

Thus far, no studies have characterized the whole transcriptomes and proteomes of porcine SPEVs. This study highlights the roles of SPEVs as important regulators of sperm motility and the roles of miR-222 as a critical EV component that participates in boar sperm function after delivery to the sperm.

Small EVs are defined as microvesicles < 200 nm in diameter (Thery et al., 2018). To evaluate the purity of EVs isolated from seminal plasma, multiple methods were employed, including TEM, NTA and Western blotting. Our results showed that the SPEV diameters of boars were concentrated in the range of 50–200 nm. The enrichment of Alix, Tsg101 and CD63 in isolated SPEVs concurred with the criteria for defining EVs.

It has been reported that SPEVs influence sperm function (Arienti et al., 1999). To determine whether EVs isolated from the seminal plasma of individuals with high vs. low sperm motility have different effects on sperm, we added such SPEVs to sperm. We found that sperm motility decreased upon the addition of SPEVs from low-sperm motility boars. To determine the differences in the contents of SPEVs from the different groups, we conducted quantitative proteomic analysis and deep RNA sequencing of SPEVs. We identified 76 DEPs from the SPEVs of boars with high- and low-sperm motility phenotypes. Thirty-one out of 76 proteins (40.7%) were predicted to be membrane proteins. In addition, 7 DEMs, 67 DELs and 126 DEGs were identified between the two groups. These results demonstrated that both the protein and nucleic acids contained in SPEVs from high- and low-sperm motility individuals were significantly different. We predicted the target genes of DEMs and found that these genes were enriched in some important signaling pathways, such as the Jak-STAT, PI3K/AKT, MAPK and apoptosis signaling pathways. Previous studies have shown that members of the Jak-STAT pathway exist and are active in sperm (D’Cruz et al., 2001; Lachance and Leclerc, 2011). The PI3K/AKT and MAPK signaling pathways have also been found to play important roles in sperm motility regulation (Fernandez et al., 2019; Yu et al., 2019) and the acrosome reaction (Kawano et al., 2010). In addition, recent studies have suggested that some miRNAs play important roles in regulating sperm apoptosis (Ma et al., 2018). Thus, we constructed a ceRNA regulatory network for five signaling pathways and found that the six DEMs interact with DELs and DEPs. Our results provide a novel perspective on the mechanisms of sperm motility.

MicroRNAs can regulate target genes at the posttranscriptional level and inhibit the expression of target genes through translational repression or degradation of nucleic acids (Bartel, 2009). Some X-linked miRNAs are considered to be critically involved in spermatogenesis and male reproduction (Song et al., 2009; Li et al., 2010; Qing et al., 2017). MicroRNA-222 is located within an X-chromosome miRNA cluster, and the miR-222 sequence is highly conserved in pigs, humans, mice, cattle and other species, suggesting that it may be related to male fertility and sperm function and that the effect may be similar across species. As a key regulatory factor, miR-222 has also been reported to be involved in many other biological processes, such as cell proliferation, cell death, autophagy and apoptosis (Wang et al., 2015; Ding et al., 2018; Gan et al., 2020). In the present study, the results suggested that miR-222 can significantly improve sperm motility. We found that there was a significant difference in the expression of miR-222 in sperm incubated with H-SPEVs compared with sperm incubated with L-SPEVs. In addition, the expression trends of EV-miR-222 in sperm incubated with the H-SPEV and L-SPEV groups varied with time. This finding indicates that miR-222 in SPEVs was transferred to sperm and that some molecules in SPEVs or sperm may affect the expression of miR-222 in sperm. The mechanism by which sperm take up EV miRNAs remains unclear and needs to be further studied.

**FIGURE 7:**
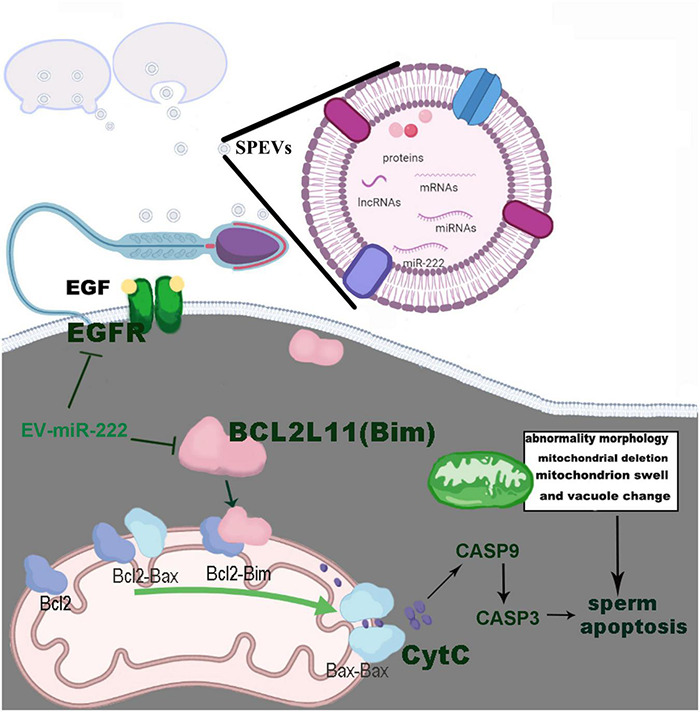
Pathways by which EV-miR-222 regulates apoptosis of porcine sperm.

To understand the effect of miR-222 on sperm morphology and function, spermatozoa incubated with SPEVs electroporated with the miR-222 mimic and inhibitor were observed by electron microscopy. We observed that a vesicle-like structure existed near the mitochondria within the cell membrane of the sperm tail. In the miR-222 inhibitor group, some mitochondrial ridges became obscure and swollen, and some mitochondria were absent. In contrast, the morphology of mitochondria was normal in the mimic group. Mitochondria play an important role in sperm motility (Amaral et al., 2013). When mitochondria are stimulated by internal and external signals, the permeability of the mitochondrial outer membrane increases, and Cyt-C (*CYCs*) in mitochondria is released into the cytoplasm (Garrido et al., 2006). The binding of Cyt-C and the apoptosis-inducing factor Apaf-1 ultimately activates caspase-3 (*CASP3*) and leads to apoptosis. Previous studies have shown that the Bcl2 family controls apoptosis by controlling mitochondrial integrity (Wang, 2001). BCL2L11, a member of the Bcl-2 family, binds to BAX on the mitochondrial membrane and activates it to promote apoptosis (Cory and Adams, 2002). Therefore, we detected the expression of the proapoptotic factors *BCL2L11*, *BAX*, *CYC*s and *CASP3* and found that these genes were significantly downregulated in the miR-222 mimic group compared with the inhibitor group. Similar results were obtained in protein expression of BCL2L11, CASP3, and CASP9. These data indicate that miR-222 decreases the binding of BCL2L11 to BAX by targeting the expression of *BCL2L11*, thus maintaining the stability of the mitochondrial membrane. In this case, mitochondrial release of Cyt-C to the cytoplasm was reduced, and the expression of Caspase-3 was inhibited, preventing sperm apoptosis (Figure 7). The mechanism by which miR-222 affects sperm function needs to be further studied.

It is worth noting that whether transcriptional or translational events exist in sperm is the focus of the recent research (Ren et al., 2017). Since condensation of chromosomes occurs in spermatids, mature sperm has been considered to be in a repressed state of transcription and translation activities. However, an increasing number of studies showed that sperm carry many different types of RNA, including mRNA, microRNA, piRNA, tRNA, rRNA, etc. (Zhao et al., 2006; Feugang et al., 2010; Card et al., 2013; Godia et al., 2019). Recently, a study reveals that mRNA are dynamically expressed in the four stages of sperm cells and several stage-specific marker genes of human germ cells were identified by using single-cell RNA sequencing (Wang et al., 2018). This study also found that the gene expression patterns in non-obstructive azoospermia patient has changed, suggesting transcriptional programs play an important role during spermatogenesis (Wang et al., 2018). Giordano and Magnano discovered that reverse transcriptase activity is present in mature spermatozoa (Giordano et al., 2000). Subsequently, it was found that the transcription and splicing activities are initiated in mature sperm (Pittoggi et al., 2006). Moreover, a comparative analysis of the transcripts between bovine spermatids and mature sperm showed that 4.5% were specific to mature sperm (Gilbert et al., 2007). Another study compared the small RNA profiles in pig seminal plasma, ejaculated sperm and epididymal sperm, and found that a large number of small RNAs from ejaculated sperm are ejaculated sperm specific (Chen et al., 2017). These data indicated that sperm may have transcriptional potential (Ren et al., 2017). Furthermore, a recent study showed that MIWI/piRNA is responsible for activating translation of spermiogenic mRNAs and revealed a critical role of the piRNA in translation activation in mouse spermatids (Dai et al., 2019).

Sperm motility is an important index to evaluate sperm fertilization ability. Many studies showed that the transcripts in sperm vary with different sperm motilities (Lambard et al., 2003; Lambard, 2004; Curry et al., 2011; Ganguly et al., 2013; Guo et al., 2017; Dorostghoal et al., 2020), indicating that some mRNA or microRNA molecules are necessary in mature sperm for continuous regulating sperm motility. For example, the expression of *ANXA2* and *BRD2* is positively correlated with the progressive motility of sperm (Jodar et al., 2012). The role of microRNA in post-transcription regulation during spermatogenesis is being paid more and more attention. A recent study showed that high levels of sperm miR-26a-5p transcript or low expression level of its target *PTEN* were associated with high sperm motility and normal morphology (Dorostghoal et al., 2020). It was also reported that the progressive sperm motility of miR-34a knockout zebrafish increased significantly (Guo et al., 2017). In addition, the expression of let-7d and let-7e were increased in sperm of boars with low sperm motility when compared to normal controls (Curry et al., 2011). In recent years, EV has been shown to play an important role in transmitting signals involved in cellular function, including germ cell maturation. Many studies have proved that EV can transfer various RNA molecules to sperm (Jodar, 2019; Chen et al., 2020). It has been suggested that EVs from seminal plasma can not only regulates a series of physiological activities of mature sperm (Arienti et al., 1999; Pons-Rejraji et al., 2011; Du et al., 2016; Murdica et al., 2019b), but also regulates the development of offspring after being absorbed by sperm (Chan et al., 2020). Thus, it is speculated that the cargo of SPEV, such as mRNAs or microRNAs, may regulate sperm motility. In the current study, some important DEM, DEG, DEL, and DEP in SPEV were identified between high- and low-sperm motility groups, suggesting that there is a link between the changes of EV cargo and sperm motility. Our experiments demonstrated that miR-222 in SPEV can be transferred to sperm *in vitro*, so as to increase the expression of miR-222 in sperm and improve sperm motility. On the other hand, the expression of its target *EGFR* and *BCL2L11* in sperm decreased significantly. The protein expression levels of EGFR and BCL2L11 in sperm of EV-miR-222 mimic group were significantly lower than that of EV-miR-222 inhibitor group. Furthermore, luciferase reporter analysis suggested that *EGFR* and *BCL2L11* are directly regulated by miR-222. These data suggested that EV-miR-222 may promote sperm motility by inhibiting the expression of EGFR and BCL2L11 in sperm and altering the gene expression in related pathways.

It has been reported that miR-222-3p expression in SPEVs is significantly different between azoospermic patients and normozoospermic individuals (Kaddour et al., 2020). Moreover, the expression of miR-222 is significantly different between PCa patients and healthy controls or individuals with benign prostatic hyperplasia (BPH) (Barcelo et al., 2019). In the present study, we compared the sequences of miR-222 and the 3′UTRs of its target genes *BCL2L11* and *EGFR* in pigs and humans and found that the sequences are highly conserved in these two species, indicating that miR-222 may play a similar role in human sperm motility. Pigs are useful biomedical models for elucidation of the mechanism of miR-222 in human sperm. Therefore, we believe that the findings of this study have implications for human sperm reproduction.

In conclusion, we report a study revealing the whole transcriptomes and proteomes of boar SPEVs. We observed that uptake of EV-miR-222 by sperm and EV-miR-222 significantly increased the sperm motility. In addition, miR-222 targeted EGFR and BCL2L11 and inhibited sperm apoptosis by reducing the expression of the proapoptotic factors BCL2L11, *BAX*, *CYC*s, CASP9 and CASP3. These findings suggest that miR-222 communicates with sperm via EVs to support sperm motility. Our study provides new insights into the molecular mechanisms by which EVs affect sperm motility.

## Data Availability Statement

The datasets presented in this study can be found in online repositories. The names of the repository/repositories and accession number(s) can be found below: The raw protein data to PRIDE with accession PXD027541 (https://www.ebi.ac.uk/pride/archive/projects/PXD027541/private) and the whole transcriptome data to NCBI and accession number is PRJNA748952 (https://www.ncbi.nlm.nih.gov/sra/PRJNA748952).

## Ethics Statement

The animal study and the research were reviewed and approved by the Committees for Ethical Review of China Agricultural University.

## Author Contributions

LJ conceived and designed the study. YD, ND, SX, and MH performed the experiments. YZ, ND, and XD analyzed the data. WD and QZ provided technical support. YD, ND, and YZ wrote the manuscript. LJ revised the manuscript. All authors read and approved the final manuscript.

## Conflict of Interest

The authors declare that the research was conducted in the absence of any commercial or financial relationships that could be construed as a potential conflict of interest.

## Publisher’s Note

All claims expressed in this article are solely those of the authors and do not necessarily represent those of their affiliated organizations, or those of the publisher, the editors and the reviewers. Any product that may be evaluated in this article, or claim that may be made by its manufacturer, is not guaranteed or endorsed by the publisher.

## Footnotes

1
http://ftp.ensembl.org/pub/release-97/fasta/sus_scrofa/

2
http://www.mirbase.org/

## Supplementary Material

Supplementary File 1 Supplementary Figures 1–5 mentioned in this study.

Supplementary File 2 Supplementary Tables 1–8 mentioned in this study.
