# Supplementary material for: MicroRNA-222 Transferred From Semen Extracellular Vesicles Inhibits Sperm Apoptosis by Targeting BCL2L11
Source: Front Cell Dev Biol. 2021 Nov 8;9:736864. doi: 10.3389/fcell.2021.736864 (PMC8607813; doi:10.3389/fcell.2021.736864)
Supplement: Supplementary File 1 — Supplementary Figures 1–5 mentioned in this study. [file Data_Sheet_1.docx]

**Supplement**

**Supplementary Figure S1**


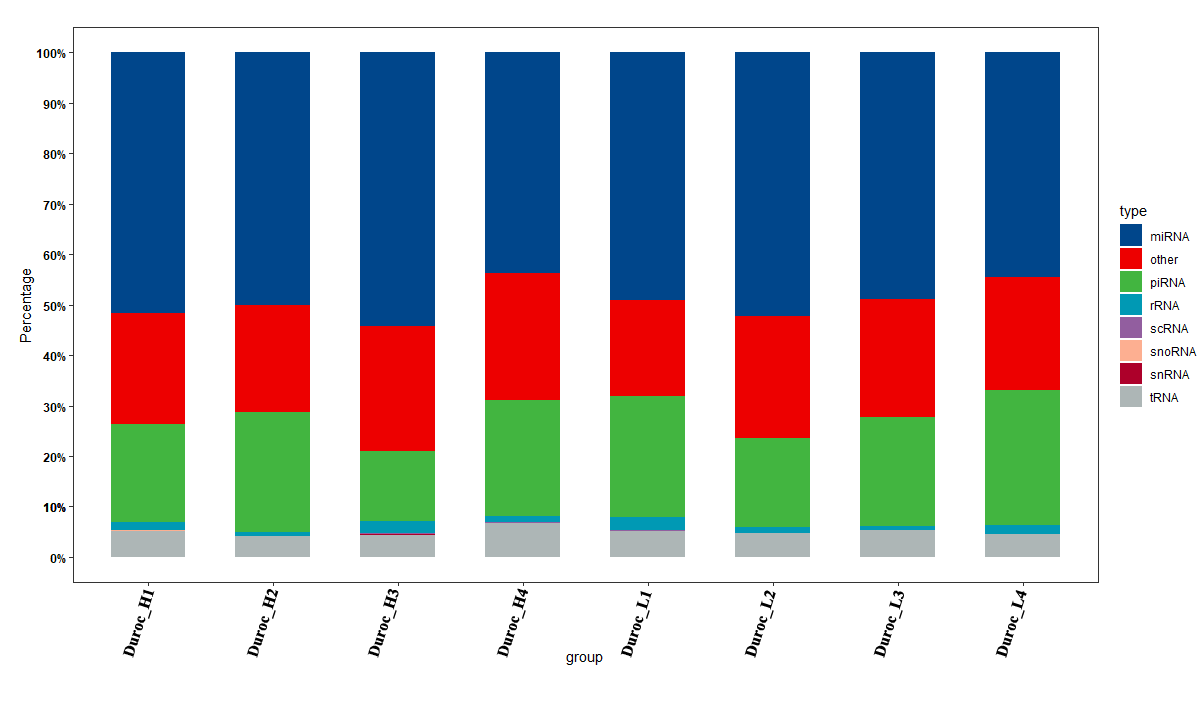


**Supplementary Figure 1. Proportion of different types of small RNAs**

**Supplementary Figure S2**


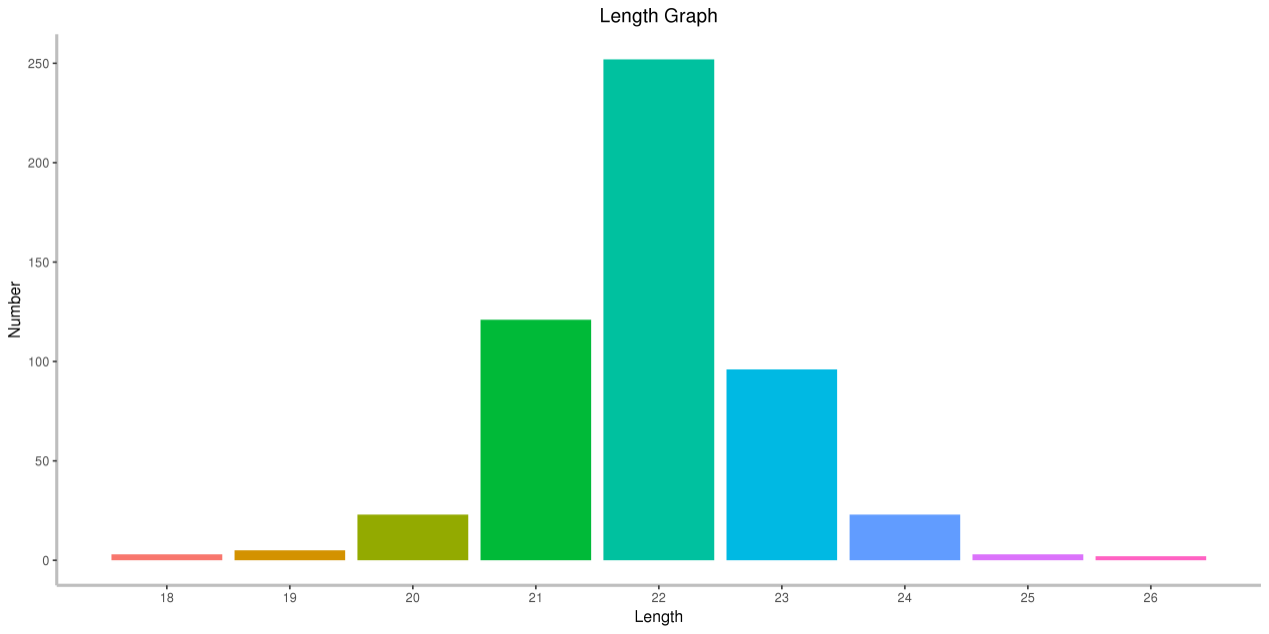


**Supplementary Figure 2. The length distribution of miRNAs in all samples**

**Supplementary Figure S3**


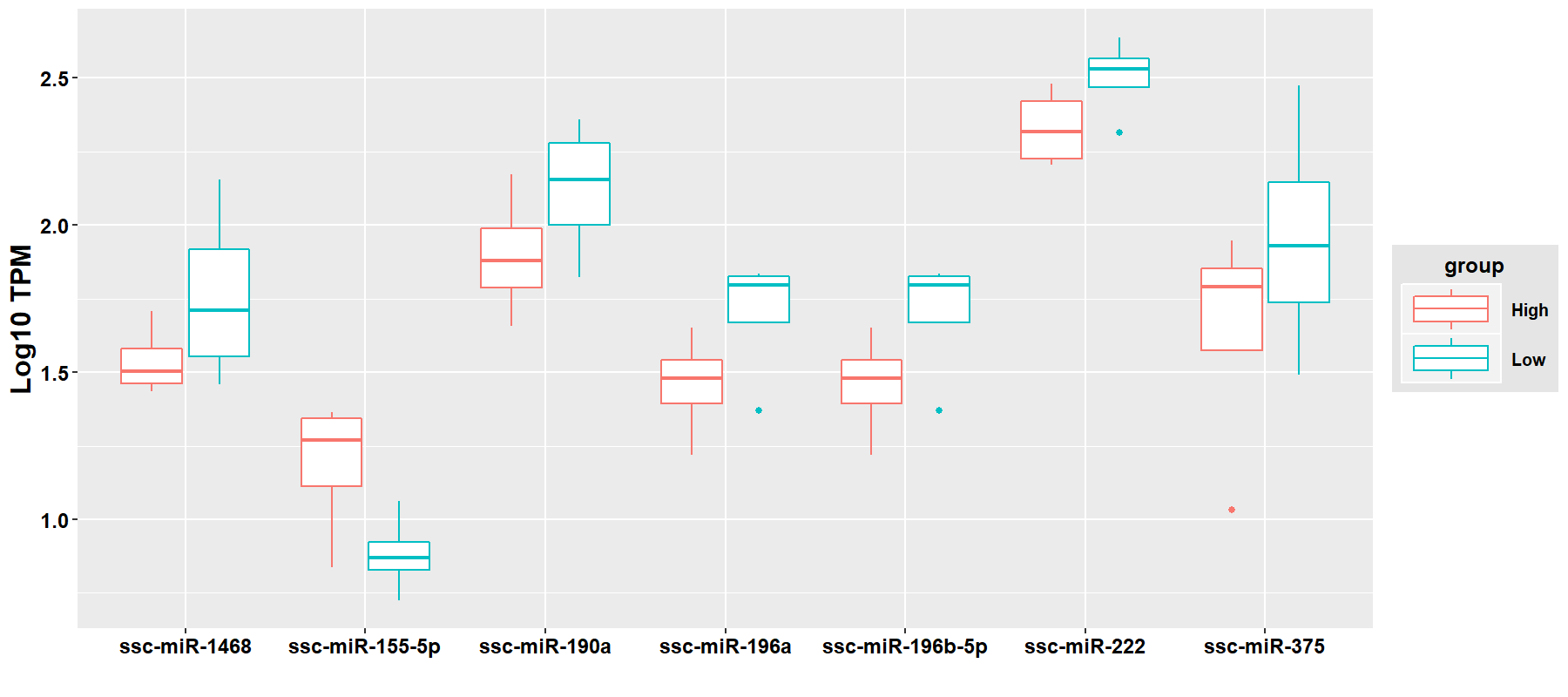


**Supplementary Figure 3. Expression levels of differentially expressed miRNAs in High and Low groups based on RNA-seq data**

**Supplementary Figure S4**


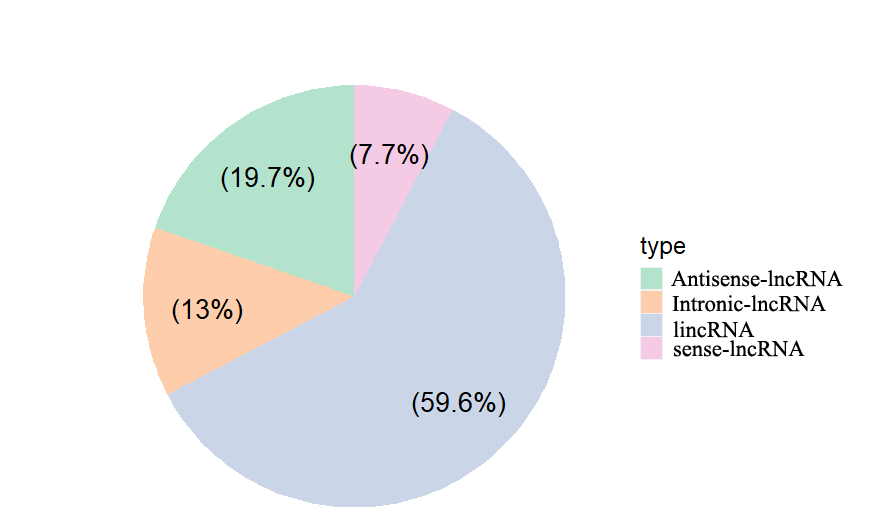


**Supplementary Figure 4. Proportion of different types of lncRNAs**

**Supplementary Figure S5**


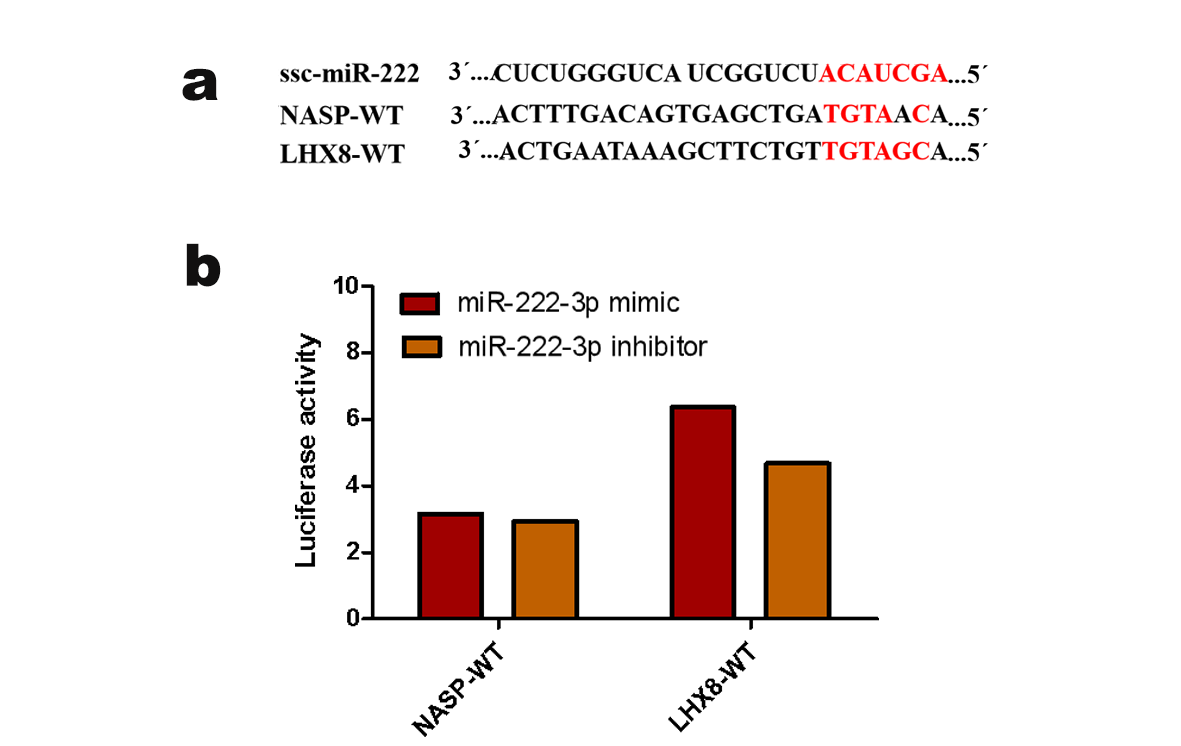


**Supplementary Figure 5. MiR-222 had no effect on the luciferase activity of wild-type *LHX8* and *NASP***

**a** The predicted target-binding sites between miR-222 and *NASP* and between miR-222 and *LHX8*. **b** A luciferase activity assay showed that miR-222 does not target the *NASP* 3'UTR and LHX8 3'UTR.
