# Supplementary material for: MicroRNA-222 Transferred From Semen Extracellular Vesicles Inhibits Sperm Apoptosis by Targeting BCL2L11
Source: Front Cell Dev Biol. 2021 Nov 8;9:736864. doi: 10.3389/fcell.2021.736864 (PMC8607813; doi:10.3389/fcell.2021.736864)
Supplement: Supplementary File 2 — Supplementary Tables 1–8 mentioned in this study. [file Data_Sheet_2.doc]

**Supplement**

| **Table S1. Information on the experimental animals** | | | |
| --- | --- | --- | --- |
| **Boar** | **Age in months** | **Sperm motility (%)** | **Fast-moving sperm (%)** |
| H1 | 15 | 99.3 | 78.4 |
| H2 | 17 | 97.2 | 79.1 |
| H3 | 14 | 99.1 | 72 |
| H4 | 22 | 96.3 | 80.4 |
| Mean±SD | 17±3.5 | 98.0±1.5 | 77.5±3.7 |
| L1 | 21 | 74.3 | 33 |
| L2 | 20 | 78.8 | 50.1 |
| L3 | 16 | 79.3 | 43 |
| L4 | 15 | 74.9 | 37.7 |
| Mean±SD | 18±2.9 | 76.8±2.6 | 41.0±7.3 |
| *P* value |  | 0.00046^***^ | 0.00014^***^ |
| ***:P<0.001 | | | |

| **Table S2. Primers used for real time qPCR** | | | | |
| --- | --- | --- | --- | --- |
| **Primer name** | **Sequence (5′→3′)** | **Length (nt)** | **GC(%)** | **Tm** |
| miR-155-5p-RT | GTCGTATCCAGTGCAGGGTCCGAGGTATTCGCACTGGATACGACCCCCTA | 50 | 58 | 87 |
| miR-155-5p-F | AGCCCGTTAATGCTAATTGTGA | 22 | 40 | 60.8 |
| miR-155-5p-R | TCGCACTGGATACGACCCCCTA | 22 | 59 | 67 |
| miR-222-RT | GTCGTATCCAGTGCAGGGTCCGAGGTATTCGCACTGGATACGACGAGACC | 50 | 58 | 87 |
| miR-222-F | ACGCAGCTACATCTGGCTACTG | 22 | 54 | 61.9 |
| miR-222-R | ATTCGCACTGGATACGACGAGACC | 24 | 54 | 67 |
| U6-RT | AACGCTTCACGAATTTGCGT | 20 | 45 | 63 |
| U6-F | CTCGCTTCGGCAGCACA | 17 | 64 | 63.6 |
| U6-R | AACGCTTCACGAATTTGCGT | 20 | 45 | 63 |
| BCl2L11-F | CAACACAAACCCCAAGTCCT | 20 | 50 | 59.9 |
| BCl2L11-R | ACTCCTGCGCAATCCATATC | 20 | 60 | 60.1 |
| EGFR-F | TGCCTTAGCCGTCTTATCCA | 20 | 50 | 60.7 |
| EGFR-R | TCGCTGTTGACAATGTCCCT | 20 | 50 | 61.7 |
| BAX-F | TTTGCTTCAGGGTTTCATCC | 20 | 45.1 | 60 |
| BAX-R | GACACTCGCTCAACTTCTTGG | 21 | 52.4 | 60 |
| CYCs-F | GAAAAGGGAGGCAAACACAA | 20 | 45 | 60 |
| CYCs-R | CCAGGTGATGCCTTTGTTCT | 20 | 50 | 60 |
| CASP3-F | GCCATGGTGAAGAAGGAAAA | 20 | 45 | 60.1 |
| CASP3-R | GGCAGGCCTGAATTATGAAA | 20 | 45 | 60 |
| MSTRG.5437.9-F | CTCTGACAACTTCAATGATGCCAG | 24 | 45 | 63.6 |
| MSTRG.5437.9-R | ACCCAGGTAGTCTTACTTCAGAGA | 24 | 45 | 58 |
| MSTRG.47157.8-F | TGAACAAACTCAGAAGCTCACT | 22 | 40 | 57 |
| MSTRG.47157.8-R | CATGCATCAATGAGGTAGAAG | 21 | 43 | 56 |
| MSTRG.6639.6-F | GCCATTTCACAGCACTGCAA | 20 | 50 | 63 |
| MSTRG.6639.6-R | ACCATCCCAAAGCGTTCCAT | 20 | 50 | 63.8 |
| MSTRG.24929.1-F | GGCTGCCTTATTTTTGTTTCCACG | 24 | 45.8 | 66.6 |
| MSTRG.24929.1-R | GTTCTCAGTTCTCAAAGTCGGGT | 23 | 47.8 | 61 |
| MSTRG.7432.12-F | GCTGATTTAGGAGGTGCAGGAT | 22 | 50 | 61 |
| MSTRG.7432.12-R | TCATCACATCCACCGGACAATT | 22 | 45.5 | 64.6 |
| GAPDH-F | TGGTGAAGGTCGGAGTGAAC | 20 | 55 | 61.1 |
| GAPDH-R | GGAAGATGGTGATGGGATTTC | 21 | 47.6 | 60.5 |
| ACTB-F | GGCATCCTGACCCTCAAGTA | 20 | 55 | 60.1 |
| ACTB-R | CACGCAGCTCGTTGTAGAAG | 20 | 55 | 59.8 |

| **Table S3. Primers used for PCR** | | | | |
| --- | --- | --- | --- | --- |
| **Primer name** | **Sequence (5′→3′)** | **Length (nt)** | **GC (%)** | **Tm** |
| w-EGFR-F | CGAGCTCAGATGTGTCCTTGGAAGT | 25 | 52 | 62 |
| w-EGFR-R | GCTCTAGAAGTGGAATCAAGTGTTAG | 26 | 42 | 60 |
| T-EGFR-F | CGAGGAAGAAGCTCACTTGTTATACTTGGGCCG | 33 | 51.5 | 70 |
| T-EGFR-R | ATAACAAGTGAGCTTCTTCCTCGTTGGAAAAGC | 33 | 42.4 | 71.4 |
| wBCL2L11-F | CGAGCTCAGTAACTTTGACTACT | 23 | 43.5 | 54.9 |
| wBCL2L11-R | GCTCTAGATTTCGGGTGTCTCTAC | 24 | 50 | 59 |
| tBCL2L11-F | TTCCTAGGACCCAGCATATGTTATATTTGTATTG | 34 | 35 | 66 |
| tBCL2L11-R | ATAACATATGCTGGGTCCTAGGAAACAAGTTAGT | 34 | 38 | 67 |

| **Table S4. Summary of read numbers based on the Small RNA-seq** | | | | | | | |
| --- | --- | --- | --- | --- | --- | --- | --- |
| **Samples** | **Raw reads** | **Clean reads** | **Annotated reads** | **Unannotated reads** | **Mapped Reads** | **Mapping ratio** | **Q30 (%)** |
| H1 | 21616667 | 18065377 | 1532250 | 16533127 | 13891436 | 84.02% | 95.62 |
| H2 | 18250626 | 15496475 | 1107778 | 14388697 | 11875614 | 82.53% | 96.16 |
| H3 | 19848044 | 16305474 | 1315403 | 14990071 | 12683204 | 84.61% | 95.47 |
| H4 | 17993588 | 13997314 | 1550741 | 12446573 | 9886696 | 79.43% | 95.47 |
| L1 | 20749226 | 17267924 | 1668488 | 15599436 | 13122744 | 84.12% | 96.2 |
| L2 | 21310754 | 18274556 | 1331606 | 16942950 | 14160319 | 83.58% | 95.62 |
| L3 | 21821253 | 18460052 | 1702462 | 16757590 | 13515182 | 80.65% | 96.17 |
| L4 | 36922802 | 28818583 | 3066819 | 25751764 | 20617082 | 80.06% | 96.03 |
| Mapping ratio=Mapped_Reads/Unannotated_reads; Annotated reads:Reads annotated as rRNA、scRNA、snRNA、snoRNA、tRNA、Repbase（repeated sequence） | | | | | | | |

| **Table S5. Summary of Long RNA libraries clean reads mapping to the reference genome sequence** | | | | | | | |
| --- | --- | --- | --- | --- | --- | --- | --- |
| **Samples** | **Reads Sum** | **Base Sum** | **Cleaned total reads** | **Mapped reads** | **Mapped Unique reads** | **Mapping ratio** | **Q30 (%)** |
| H1 | 84120421 | 23911296827 | 168240842 | 138312522 | 91061054 | 82.21% | 90.59 |
| H2 | 84140408 | 24547085804 | 168280816 | 145523195 | 94172797 | 86.48% | 91.79 |
| H3 | 99816944 | 28902150299 | 199633888 | 176499444 | 134620648 | 88.41% | 91.98 |
| H4 | 88634064 | 25841522299 | 177268128 | 147610338 | 102430377 | 83.27% | 92.67 |
| L1 | 87316526 | 25373025598 | 174633052 | 155228753 | 112730985 | 88.89% | 92.45 |
| L2 | 83626842 | 24171862756 | 167253684 | 145902930 | 90664370 | 87.23% | 92.59 |
| L3 | 81689170 | 23635961275 | 163378340 | 140450738 | 90412835 | 85.97% | 92.37 |
| L4 | 93866847 | 27062486740 | 187733694 | 155717528 | 114780768 | 82.95% | 91.98 |
| Mapping ratio=Mapped reads/Cleaned total reads; Mapped Unique reads: only one position matching reads in the genome | | | | | | | |

| **Table S6. Details of differentially expressed genes between H group and L group** | | | | | | | |
| --- | --- | --- | --- | --- | --- | --- | --- |
| **Gene name** | **Location** | **Strand** | **Average FPKM (L)** | **Average FPKM (H)** | **Log2FC** | ***P*** value | **Regulated** |
| PVALB | 5:10955491-10973289 | + | 5.46891725 | 13.2505905 | 1.856338678 | 0.000206803 | up |
| RXYLT1 | 5:28247412-28276273 | + | 4.146953 | 9.12346225 | 1.204029303 | 0.031794361 | up |
| ENSSSCG00000000550 | 5:46244221-46279077 | - | 5.708076 | 10.809021 | 1.309244657 | 0.033608321 | up |
| ACRBP | 5:64027876-64047847 | + | 9.784318 | 15.23200625 | 0.929833087 | 0.041828188 | up |
| TXNRD1 | 5:80168523-80288075 | - | 5.22328875 | 8.6395245 | 0.786868069 | 0.042114546 | up |
| LRRIQ1 | 5:96801326-97093718 | - | 4.45549525 | 13.1298175 | 1.530520904 | 0.003681151 | up |
| ZKSCAN8 | 7:21981150-22000819 | + | 3.83053175 | 9.551831 | 1.298878064 | 0.014106765 | up |
| GPX6 | 7:22256038-22267992 | - | 21.276805 | 6.90209175 | -1.50890198 | 0.000359718 | down |
| GPX5 | 7:22289407-22300727 | + | 6.22626 | 15.72591475 | 1.527921071 | 0.000719351 | up |
| LY6G5C | 7:23791239-23795734 | - | 20.86596025 | 67.4285805 | 1.929581341 | 8.87665E-05 | up |
| TTC6 | 7:62227849:62444307 | - | 5.3235065 | 14.55258075 | 1.277782668 | 0.030367871 | up |
| RNASE10 | 7:78391458-78396857 | - | 13.30227225 | 48.526511 | 1.944071666 | 6.20635E-05 | up |
| BBOF1 | 7:97316711-97368755 | + | 4.52825475 | 7.01944275 | 1.12192099 | 0.044763045 | up |
| ZC2HC1C | 7:98265274-98273353 | + | 5.697786 | 9.3344105 | 1.221154654 | 0.045130216 | up |
| ENSSSCG00000002400 | 7:100449583-100463614 | + | 4.7881595 | 8.003637 | 1.018018911 | 0.036438253 | up |
| DNAAF1 | 6:4457291-4479828 | - | 10.528309 | 24.11929625 | 1.500562327 | 0.009509435 | up |
| GGN | 6:47297572-47301553 | - | 3.05168 | 8.18310875 | 1.860615012 | 0.013125682 | up |
| SYNGR4 | 6:53784409-53793725 | + | 3.0482625 | 8.816963 | 1.762587615 | 0.019138723 | up |
| EYA3 | 6:85095409-85185799 | - | 8.7526435 | 5.36637525 | -0.815347428 | 0.047559276 | down |
| KIAA1328 | 6:120670333-121035937 | + | 4.9627525 | 11.7757595 | 1.517319416 | 0.003227166 | up |
| UTRN | 1:20511240-21048787 | - | 13.991735 | 3.80020275 | -0.949398683 | 0.02743833 | down |
| ENSSSCG00000004415 | 1:75229861-75431569 | + | 7.17401025 | 15.0587795 | 1.028241251 | 0.036445624 | up |
| COX7A2 | 1:90696698-90703410 | + | 53.45098875 | 79.351022 | 0.859662789 | 0.042951135 | up |
| LRRC49 | 1:168544421-168736644 | + | 25.971496 | 48.70593775 | 1.171827262 | 0.039006076 | up |
| C14orf39 | 1:189407491-189471537 | - | 7.44027875 | 18.21988 | 1.501709814 | 0.005395924 | up |
| CNTLN | 1:205370499-205697768 | - | 3.56399925 | 7.328042 | 1.324163145 | 0.008302688 | up |
| ANGPT1 | 4:29933135-30233960 | + | 6.82958325 | 15.71313725 | 1.300360406 | 0.04036929 | up |
| ENSSSCG00000006101 | 4:42249512-42369447 | + | 3.2892115 | 7.34705525 | 1.416743403 | 0.004488329 | up |
| CFAP45 | 4:90593858-90627809 | + | 5.780226 | 12.5975705 | 1.252765869 | 0.01336549 | up |
| CRABP2 | 4:93388092-93394155 | + | 11.5730995 | 5.178071 | -1.285058773 | 0.044381749 | down |
| UBAP2L | 4:95471126-95520425 | - | 298.2853973 | 37.2773395 | -1.574928583 | 0.015054654 | down |
| ANKRD35 | 4:99442901-99460869 | + | 12.607131 | 59.402487 | 1.359180373 | 0.017422822 | up |
| CCDC18 | 4:123861797-124009538 | - | 26.40610675 | 57.492711 | 1.199873895 | 0.020339713 | up |
| ENSSSCG00000007001 | 17:47835-217763 | - | 2.94048175 | 8.230295 | 1.735523604 | 0.008078258 | up |
| CST11 | 17:30414051-30417127 | - | 33.62710875 | 59.11943075 | 1.294976939 | 0.017046756 | up |
| ENSSSCG00000007123 | 17:30422929-30426682 | + | 3.06037225 | 10.909815 | 2.15909095 | 0.030356337 | up |
| ENSSSCG00000007219 | 17:35167886-35173747 | - | 13.8831635 | 46.137789 | 2.100256095 | 0.004793552 | up |
| SYCP2 | 17:59877505-59955209 | - | 9.39072325 | 24.16910425 | 1.244034965 | 0.018911101 | up |
| ENSSSCG00000007848 | 3:24609167-24665677 | - | 9.81944275 | 26.35863925 | 1.776929782 | 0.000644818 | up |
| REXO5 | 3:25177693-25238998 | - | 7.575439 | 16.35129875 | 1.084968601 | 0.031144861 | up |
| PPL | 3:37460502-37511876 | + | 8.73777 | 3.26324875 | -1.041342234 | 0.024480391 | down |
| MRPS34 | 3:40193232-40196868 | + | 25.1273645 | 37.77068575 | 0.921342364 | 0.023585194 | up |
| CCDC88A | 3:85811106-85941868 | + | 3.36422725 | 6.98015425 | 1.083503654 | 0.040392469 | up |
| SRD5A2 | 3:107839964-107918350 | + | 5.99542925 | 9.97967925 | 1.110680432 | 0.036522502 | up |
| DRC1 | 3:112581770-112621601 | - | 3.37761575 | 7.34956825 | 1.257640843 | 0.044105724 | up |
| LRPAP1 | 8:2125405-2145647 | - | 8.491837 | 12.45652325 | 0.90684747 | 0.045064268 | up |
| CCDC158 | 8:71990113-72088968 | - | 5.90096925 | 10.31874925 | 0.906598661 | 0.048719261 | up |
| RBM46 | 8:74311884-74382516 | - | 11.16408325 | 28.7599845 | 1.554503973 | 0.00202807 | up |
| ETNPPL | 8:113363049-113384783 | + | 9.2284515 | 2.4844965 | -1.527099619 | 0.026390292 | down |
| PIWIL1 | 14:24730053-24760013 | - | 4.3969585 | 8.84892125 | 1.425431406 | 0.019333822 | up |
| ENSSSCG00000010172 | 14:59112899-59168395 | + | 13.5703745 | 7.63503775 | -0.963267535 | 0.018328854 | down |
| PTPN20 | 14:88764980-88838942 | - | 3.90171725 | 10.02295825 | 1.777968672 | 0.011600126 | up |
| ENSSSCG00000010498 | 14:107417054-107426067 | + | 4.08936025 | 9.13371075 | 1.226720252 | 0.034458218 | up |
| SYCE1 | 14:141725884-141735864 | - | 8.59092675 | 16.760881 | 1.272364049 | 0.02987677 | up |
| SCCPDH | 10:15205648-15264489 | - | 8.3096005 | 12.230667 | 0.860695262 | 0.047257948 | up |
| CCDC39 | 13:118626329-118682283 | - | 4.17779825 | 8.8695685 | 1.093417979 | 0.030298629 | up |
| PRPS2 | X:9502748-9600301 | + | 290.7203828 | 76.4012315 | -1.765802885 | 0.024724034 | down |
| TLR7 | X:9573178-9576987 | + | 8.96820075 | 71.1146135 | 2.925768791 | 0.000369899 | up |
| DOCK11 | X:97068355-97267517 | + | 2.5365015 | 7.98690975 | 1.40544032 | 0.016495152 | up |
| MBNL3 | X:108489557-108612875 | - | 6.88815075 | 4.1706065 | -1.28558094 | 0.01497324 | down |
| ENSSSCG00000013341 | 2:34931115-35463811 | - | 3.714456 | 7.92089525 | 1.3951406 | 0.020316951 | up |
| LDHC | 2:40748863-40809910 | - | 5.96587875 | 13.65624125 | 1.437710711 | 0.0441032 | up |
| NFIX | 2:65954244-66056567 | - | 7.79099125 | 3.02691875 | -1.02680532 | 0.027962817 | down |
| COMP | 2:59061420-59068949 | + | 4.8698375 | 11.76622 | 1.450045939 | 0.007556136 | up |
| FBXL17 | 2:112989145-113480210 | - | 9.06657475 | 4.57932025 | -1.127188269 | 0.007649159 | down |
| CEP164 | 9:44670874-44752473 | + | 7.20753175 | 8.14758275 | 0.950548812 | 0.023346557 | up |
| NEK2 | 9:131547084-131560527 | + | 4.998787 | 8.07591775 | 1.18723974 | 0.043553246 | up |
| CCDC148 | 15:65062034-65431023 | - | 3.599709 | 6.569801 | 1.333627491 | 0.036595733 | up |
| SMARCD3 | 18:5958481-5990025 | + | 3.968959 | 6.0765815 | 1.226305511 | 0.046816074 | up |
| PGAM2 | 18:48693882-48713862 | + | 4.89044125 | 7.94242875 | 1.101491505 | 0.029367942 | up |
| MED10 | 16:75516439:75525361 | + | 5.42349075 | 12.04478975 | 1.226568596 | 0.045819708 | up |
| ENSSSCG00000017213 | 12:6139428-6158450 | + | 6.414815 | 11.46638025 | 1.058983327 | 0.038165299 | up |
| ENSSSCG00000017226 | 12:6353687-6368985 | + | 6.92846725 | 11.79133075 | 0.987826922 | 0.030928888 | up |
| DCAKD | 12:18203890-18365138 | + | 11.45626575 | 5.95514375 | -0.921344185 | 0.035654309 | down |
| ATF7IP2 | 3:32523461-32598855 | - | 35.9555185 | 94.9751965 | 1.523582575 | 0.016808363 | up |
| ADA | 17:47044495-47072245 | - | 55.05837875 | 27.80130575 | -1.014610008 | 0.041888289 | down |
| CCDC66 | 13:38438701-38492439 | + | 5.5476535 | 9.77472325 | 0.812398278 | 0.039705425 | up |
| ENSSSCG00000021473 | 17:8946897-8975696 | - | 2.871984 | 8.230305 | 1.869344445 | 0.00298716 | up |
| CCT6B | 12:40205121-40473528 | + | 9.2654875 | 15.2024955 | 1.145331858 | 0.035895658 | up |
| WDR78 | 6:145680052-145768488 | + | 9.24103725 | 18.589695 | 1.14232092 | 0.044213829 | up |
| KANSL1L | 15:112763186-112916642 | - | 71.9905455 | 144.865409 | 1.295111416 | 0.014430857 | up |
| MCCD1 | 7:23656873-23657999 | + | 15.750867 | 37.72152625 | 1.273317184 | 0.041054274 | up |
| TIA1 | 3:72240515-72277320 | + | 7.8070005 | 16.898977 | 1.242636826 | 0.01341349 | up |
| DDIT4L | 8:120371538-120376441 | + | 11.03226775 | 3.86911975 | -1.174331819 | 0.032188163 | down |
| CLGN | 8:86621178-86675796 | + | 7.68162725 | 12.27893675 | 1.140405671 | 0.037075297 | up |
| MAN2B2 | 8:4129643-4181212 | + | 32.672 | 52.88000175 | 1.192739436 | 0.026221961 | up |
| FAM76A | 6:84880035-84910384 | + | 9.846129 | 2.91858175 | -0.798093586 | 0.042748199 | down |
| ENSSSCG00000028629 | 17:35154518-35155738 | - | 2.2393135 | 11.7726355 | 2.697423055 | 0.000280869 | up |
| ENSSSCG00000029289 | 17:30451711-30454677 | - | 3.252947 | 10.9456565 | 1.846160221 | 0.017155494 | up |
| SPATA1 | 6:129205499-129247246 | - | 9.95388125 | 26.10814475 | 1.643609269 | 0.00225185 | up |
| DEFB124 | 17:35199826:35205172 | - | 42.6669895 | 100.859808 | 1.418553275 | 0.004717411 | up |
| AKAP12 | 1:14908115-15017001 | - | 8.071328 | 18.49046125 | 1.149756182 | 0.036334872 | up |
| CMTM2 | 6:27282070-27295522 | + | 1.56517525 | 9.08131575 | 3.051524093 | 0.000290568 | up |
| SLC39A10 | 15:99584839-99726404 | + | 2.931616 | 10.15298075 | 1.255866597 | 0.026014034 | up |
| SMC1B | 5:3976140-4067395 | + | 5.16113625 | 10.3674735 | 1.332805971 | 0.015158715 | up |
| CIAO2B | 6:27610470-27612592 | - | 4.1871355 | 11.35609875 | 1.49610194 | 0.044205475 | up |
| CYB5B | 6:17519289-17645386 | - | 19.299723 | 12.23775175 | -1.640958319 | 8.1286E-05 | down |
| KPNA6 | 6:88611462-88656278 | + | 9.406507 | 6.273907 | -0.943264236 | 0.028676782 | down |
| ENSSSCG00000034490 | 7:43999267-44000238 | - | 4.34112825 | 9.34330125 | 1.462401481 | 0.01318454 | up |
| ENSSSCG00000035210 | 15:38206311-38210659 | + | 10.56568975 | 28.024896 | 1.595665488 | 0.000456712 | up |
| DEFB127 | 17:34945498-34975946 | - | 5.3778155 | 16.56691375 | 1.716970809 | 0.01162395 | up |
| ENSSSCG00000036138 | 9:52871556-52888324 | + | 6.71027075 | 12.6790395 | 1.205129684 | 0.004871581 | up |
| SLC17A2 | 7：20596549-20663423 | - | 6.695506 | 20.351451 | 1.243014113 | 0.005639639 | up |
| ENSSSCG00000036946 | 4:99168921:99190581 | + | 16.5312805 | 3.97067275 | -2.264592247 | 0.020145569 | down |
| NUP210L | 4:95573722-95697196 | + | 29.0125445 | 64.0146435 | 1.176467347 | 0.032832558 | up |
| DKKL1 | 6:54463759-54468361 | + | 14.036415 | 21.964193 | 1.181398596 | 0.036601836 | up |
| CCDC178 | 6:116764199-117216500 | - | 3.73592025 | 9.31494425 | 1.585175836 | 0.003151709 | up |
| ENSSSCG00000039455 | 15:38166635-38171017 | - | 15.332676 | 49.20466725 | 1.660841859 | 0.00028029 | up |
| SLC2A5 | 6:69507825-69535372 | - | 4.290406 | 6.141068 | 1.335787621 | 0.039321938 | up |
| TFAM | 14:92259403-92279550 | - | 12.17674075 | 6.649738 | -0.806087682 | 0.042077731 | down |
| ECHO1907-A59-lncRNA_newGene_4200 | 13:207643401-207699957 | + | 25.97256875 | 41.5372915 | 1.09364985 | 0.049850593 | up |
| ECHO1907-A59-lncRNA_newGene_9749 | 17:3794228-3801963 | - | 0.861008 | 58.85754325 | 6.431391663 | 0.039610731 | up |
| ECHO1907-A59-lncRNA_newGene_13520 | 2:122627833-122816172 | - | 4.35607225 | 14.39879525 | 1.725953694 | 0.012178667 | up |
| ECHO1907-A59-lncRNA_newGene_20573 | 6:10762514-10961560 | + | 18.69487175 | 6.33264425 | -1.499862007 | 0.009372348 | down |
| ECHO1907-A59-lncRNA_newGene_21408 | 6:55033229-55050374 | - | 1.650167 | 8.82211175 | 2.043374021 | 0.004297895 | up |
| ECHO1907-A59-lncRNA_newGene_21549 | 6:60553319-60572501 | + | 7.34094775 | 21.70570975 | 1.367999872 | 0.041378863 | up |
| ECHO1907-A59-lncRNA_newGene_22653 | 6:128398409-128454403 | - | 5.5731745 | 11.47252075 | 1.403589838 | 0.010409134 | up |
| ECHO1907-A59-lncRNA_newGene_24430 | 7:62014473-62374220 | - | 3.9605105 | 12.91664 | 2.281827203 | 0.000525956 | up |
| ECHO1907-A59-lncRNA_newGene_29245 | AEMK02000153.1:26964-82766 | - | 9.58423725 | 13.14459325 | 0.993862413 | 0.037233918 | up |
| ECHO1907-A59-lncRNA_newGene_29343 | AEMK02000220.1:15331-17680 | + | 91.607768 | 159.0399943 | 1.202537411 | 0.045667998 | up |
| ECHO1907-A59-lncRNA_newGene_30285 | AEMK02000495.1:3912-41862 | - | 2.988541 | 7.7307785 | 1.668856246 | 0.010158195 | up |
| ECHO1907-A59-lncRNA_newGene_30286 | AEMK02000496.1:2784-128296 | - | 6.4059765 | 13.69966775 | 1.352385672 | 0.036027148 | up |
| ECHO1907-A59-lncRNA_newGene_30422 | AEMK02000556.1:37820-40186 | + | 5.1658895 | 16.56808575 | 1.931500733 | 0.007817295 | up |
| ECHO1907-A59-lncRNA_newGene_30771 | AEMK02000682.1:710882-712520 | - | 23.343712 | 46.49043275 | 1.245777734 | 0.00325008 | up |
| ECHO1907-A59-lncRNA_newGene_30835 | AEMK02000682.1:1045371-1049196 | + | 7.11801475 | 21.552669 | 1.747654705 | 0.001754411 | up |
| ECHO1907-A59-lncRNA_newGene_33175 | 10:24956931-24976515 | - | 4.61762175 | 12.2187625 | 1.627718393 | 0.03930694 | up |

| **Table S7. Details of differentially expressed lncRNAs between H group and L group** | | | | | | | |
| --- | --- | --- | --- | --- | --- | --- | --- |
| **ID** | **Location** | **Strand** | **Average FPKM (L)** | **Average FPKM (H)** | **Log2FC** | ***P* value** | **Regulated** |
| MSTRG.11174.4 | 12:19660899-19714873 | - | 0.033122 | 5.009913 | 7.601698583 | 0.017656477 | up |
| MSTRG.11174.6 | 12:19671179-19714873 | - | 3.72799475 | 0.00854875 | -7.988685945 | 0.011967205 | down |
| MSTRG.13242.2 | 13:13872360-13876301 | + | 17.55305475 | 0 | -12.28177999 | 4.62469E-06 | down |
| MSTRG.15790.1 | 13:120586730-120711683 | + | 5.03451675 | 0.01665625 | -7.673412105 | 0.020335993 | down |
| MSTRG.16164.2 | 13:133969455-133970536 | - | 0.04763575 | 2.4589385 | 5.919395506 | 0.039723862 | up |
| MSTRG.18947.2 | 14:40286506-40310606 | - | 7.03748125 | 0.2404385 | -4.354899621 | 0.044055709 | down |
| MSTRG.20555.61 | 14:92532500-92646242 | + | 0.03061925 | 2.885888 | 5.735324852 | 0.049634817 | up |
| MSTRG.20603.5 | 14:93566856-93658947 | - | 0 | 0.80151625 | 10.06810179 | 0.001373951 | up |
| MSTRG.21268.16 | 14:115179120-115201958 | - | 0.01055525 | 3.8711415 | 7.828707781 | 0.002565597 | up |
| MSTRG.22063.1 | 15:3267817-3271113 | + | 0.007073 | 0.83374175 | 5.321663601 | 0.040851768 | up |
| MSTRG.24929.1 | 15:134623023-134623835 | + | 0.00205025 | 1.51274625 | 8.586222972 | 0.002381742 | up |
| MSTRG.25344.1 | 16:8654524-8688976 | - | 0 | 0.412635 | 7.723890139 | 0.007795956 | up |
| MSTRG.26112.1 | 16:39958522-40076869 | + | 3.434654 | 0.003862 | -8.954749676 | 0.002131829 | down |
| MSTRG.27323.1 | 17:11551408-11613936 | - | 0.02979425 | 13.643346 | 8.069867934 | 0.01524454 | up |
| MSTRG.29633.2 | 18:19115706-19140325 | + | 15.8751295 | 1.14894775 | -3.566117227 | 0.024723901 | down |
| MSTRG.30656.1 | 18:55883918-55889341 | + | 0.00594625 | 1.54065325 | 7.074658938 | 0.016720046 | up |
| MSTRG.32134.1 | 2:43388321-43723127 | - | 0.76383525 | 0.005479 | -7.118150453 | 0.037070252 | down |
| MSTRG.34506.5 | 2:124839984-125173642 | + | 0.655139 | 0.00745025 | -6.501725119 | 0.048308371 | down |
| MSTRG.34506.8 | 2:124856426-125214256 | + | 3.72128075 | 0.020748 | -6.945576875 | 0.001692875 | down |
| MSTRG.34931.1 | 2:142177330-142189362 | + | 0.34291925 | 51.942916 | 6.84878517 | 0.008224969 | up |
| MSTRG.36572.1 | 3:30028908-30031203 | + | 0.752139 | 0 | -9.41741066 | 0.006236516 | down |
| MSTRG.36663.1 | 3:33552275-33554074 | - | 0.59691475 | 0.00741575 | -5.367993206 | 0.048298875 | down |
| MSTRG.36807.3 | 3:38551364-38562903 | + | 18.4055765 | 0.846908 | -4.393680948 | 0.032184879 | down |
| MSTRG.39520.1 | 3:127697252-127724762 | + | 0 | 0.14660425 | 5.443441374 | 0.034399183 | up |
| MSTRG.40653.3 | 4:41209264-41226170 | - | 0.0447715 | 2.2224385 | 5.521437149 | 0.045997304 | up |
| MSTRG.41456.1 | 4:75686527-75757005 | - | 1.5445495 | 0.0067905 | -7.519716859 | 0.006173753 | down |
| MSTRG.43085.3 | 4:120722401-120764451 | - | 9.79979975 | 0.13947675 | -6.422129948 | 0.025566696 | down |
| MSTRG.44534.1 | 5:22153540-22178115 | - | 9.4692845 | 0 | -11.46983554 | 1.6845E-05 | down |
| MSTRG.4463.1 | 1:194846823-194861936 | + | 0 | 0.773911 | 9.351553029 | 0.004081407 | up |
| MSTRG.45076.2 | 5:45749295-45751096 | - | 0.98413125 | 0.000016 | -8.068034941 | 0.002219576 | down |
| MSTRG.46269.39 | 5:86104259-86239302 | - | 0.26940025 | 0.00141775 | -6.874556966 | 0.02299668 | down |
| MSTRG.46376.14 | 5:89728415-89954362 | + | 0 | 3.5428335 | 10.87222209 | 0.000357654 | up |
| MSTRG.46376.15 | 5:89728415-90088263 | + | 0 | 0.8839995 | 9.439680711 | 0.003923611 | up |
| MSTRG.47157.8 | 6:10762506-10822464 | + | 0.0072835 | 0.28934125 | 4.879683281 | 0.024664215 | up |
| MSTRG.48589.1 | 6:50275524-50279085 | - | 0.002525 | 1.62932175 | 9.209088985 | 0.004269766 | up |
| MSTRG.48892.1 | 6:54016892-54023265 | - | 0.00011925 | 0.423287 | 6.677098165 | 0.016576721 | up |
| MSTRG.49186.2 | 6:58550401-58556493 | - | 0.01182825 | 3.165229 | 7.210127922 | 0.003874731 | up |
| MSTRG.49319.4 | 6:60558765-60572078 | + | 0.0932425 | 12.423555 | 6.487127524 | 0.017582387 | up |
| MSTRG.49890.4 | 6:71666816-71675022 | + | 19.08351625 | 1.19913125 | -3.941559207 | 0.013861582 | down |
| MSTRG.51314.1 | 6:112744123-112754691 | + | 0 | 2.914543 | 9.392030188 | 0.006030198 | up |
| MSTRG.5437.9 | 1:241441984-241551191 | - | 0 | 0.29266975 | 7.580706589 | 0.006073338 | up |
| MSTRG.54838.1 | 7:55912265-55918711 | + | 0.01261025 | 0.90882575 | 5.205152426 | 0.038407504 | up |
| MSTRG.55869.1 | 7:91760592-91785276 | - | 0.219061 | 7.2350925 | 5.411804491 | 0.021583704 | up |
| MSTRG.57210.1 | 8:15970819-15987912 | + | 1.4600745 | 0.0477445 | -5.266474011 | 0.025466742 | down |
| MSTRG.57499.1 | 8:32088801-32126850 | + | 0.38247375 | 9.926511 | 4.228929715 | 0.020166789 | up |
| MSTRG.57820.1 | 8:47734487-47757755 | + | 0.01590975 | 3.369071 | 6.683019709 | 0.03937967 | up |
| MSTRG.58514.1 | 8:82322152-82327266 | - | 0 | 0.50031725 | 9.267440858 | 0.004956899 | up |
| MSTRG.59277.1 | 8:120038088-120089700 | - | 0 | 0.714492 | 9.343283814 | 0.003551608 | up |
| MSTRG.60775.29 | 9:37936997-37938088 | + | 0 | 3.71935825 | 10.76306187 | 0.00081805 | up |
| MSTRG.60818.1 | 9:39082906-39086065 | + | 1.79863475 | 0.02928525 | -5.662891874 | 0.04008912 | down |
| MSTRG.61770.1 | 9:72305478-72325428 | - | 1.0298505 | 0.0163895 | -5.110527779 | 0.048738154 | down |
| MSTRG.61778.1 | 9:72392893-72399076 | + | 2.35017375 | 0.005068 | -7.662636593 | 0.007540905 | down |
| MSTRG.63366.4 | AEMK02000135.1:6616-8732 | - | 0.07780825 | 5.90086575 | 6.554490656 | 0.034055492 | up |
| MSTRG.63481.3 | AEMK02000218.1:101-2118 | + | 0.11777625 | 1.87083525 | 4.100633049 | 0.045953999 | up |
| MSTRG.64254.1 | AEMK02000452.1:2892320-2895639 | + | 0 | 2.200078 | 9.004172721 | 0.007700589 | up |
| MSTRG.65285.1 | X:11168892-11279977 | - | 0.011142 | 0.77421 | 5.813705222 | 0.035200601 | up |
| MSTRG.65860.2 | X:40353719-40561812 | - | 0 | 0.5693685 | 8.277624955 | 0.007137442 | up |
| MSTRG.6603.1 | 10:7715692-7718052 | - | 0.014201 | 1.235263 | 6.118743444 | 0.014804196 | up |
| MSTRG.6639.6 | 10:8551926-8559252 | + | 0.00306175 | 0.23182225 | 6.155212939 | 0.025618792 | up |
| MSTRG.67594.9 | X:112011363-112043984 | + | 5.7258535 | 0.0469345 | -6.410415073 | 0.01292206 | down |
| MSTRG.68105.2 | Y:25273275-25273680 | - | 0.42830675 | 77.7042605 | 7.939181845 | 0.019084953 | up |
| MSTRG.7061.1 | 10:20013073-20022191 | + | 6.0079105 | 0.01998625 | -7.637032281 | 0.007062979 | down |
| MSTRG.7291.6 | 10:28763402-28770684 | + | 17.2748695 | 0.35299025 | -6.114333727 | 0.030338175 | down |
| MSTRG.7432.12 | 10:33561497-33683886 | + | 0 | 1.383865 | 9.824886857 | 0.003487739 | up |
| MSTRG.7455.17 | 10:35404862-35448229 | + | 0 | 0.8021075 | 7.920756776 | 0.008425965 | up |
| MSTRG.8652.1 | 11:4760357-4840544 | + | 1.22129375 | 0 | -6.613431218 | 0.020069096 | down |
| MSTRG.9664.1 | 11:51781790-51784280 | + | 10.204427 | 0.16280075 | -6.047487799 | 0.01070016 | down |

| **Table S8. Details of differentially expressed proteins between H group and L group** | | | | | | |
| --- | --- | --- | --- | --- | --- | --- |
| **Accession** | **Gene Name** | **H Group average** | **L Group average** | **Log2FC** | ***P* value** | **Regulated** |
| A0A287AKL9 | NA | 2810586.799 | 497419.3741 | 2.498336765 | 0.018260644 | up |
| F1RWV2 | TIMP1 | 11092578.65 | 1972975.292 | 2.491149988 | 0.025165583 | up |
| A0A287AZU8 | MAN2C1 | 2041439.259 | 383444.5582 | 2.412496742 | 0.002924172 | up |
| P00999 | NA | 1461285.713 | 357000.439 | 2.03324053 | 0.024574187 | up |
| F1RM87 | ADAM28 | 133782925.4 | 33559850.46 | 1.995085806 | 0.022733906 | up |
| F1RK12 | ITM2B | 5137965.265 | 1311133.468 | 1.970382583 | 0.037124993 | up |
| I3LT50 | ESPN | 13038938.77 | 3543051.202 | 1.879762234 | 0.023900998 | up |
| A0A286ZKS6 | HGS | 8345202.785 | 2430142.492 | 1.779906198 | 0.005632786 | up |
| A0A287AP77 | MVB12A | 3355608.725 | 987401.3379 | 1.764865998 | 0.02722557 | up |
| F1S5H7 | NHLRC2 | 248222.5681 | 81486.04546 | 1.607009366 | 0.036230031 | up |
| F6Q109 | GLUL | 12025734.77 | 3998388.345 | 1.588634539 | 0.046289536 | up |
| F1S6Z9 | ADGRG6 | 12400084.93 | 4325168.419 | 1.519521786 | 0.006331261 | up |
| A0A286ZTB0 | SELENBP1 | 5582405.9 | 2097668.854 | 1.412100081 | 0.018272317 | up |
| F1RM85 | ADAM7 | 111137117.8 | 42601048.97 | 1.383379872 | 0.002630976 | up |
| K7GLP9 | SMS | 3112424.036 | 1201186.799 | 1.373578101 | 0.039009998 | up |
| A0A1P8VJR2 | APN | 113082731.3 | 44594316.78 | 1.342446868 | 0.039515321 | up |
| Q06AU4 | RAB34 | 1142294.862 | 453941.2567 | 1.331357584 | 0.004266408 | up |
| A0A1X9RRB4 | EPCAM | 19783134.27 | 7870530.52 | 1.329738222 | 0.039288264 | up |
| Q5K4Q3 | SWC3 | 590593.2186 | 252869.3645 | 1.223772528 | 0.028609668 | up |
| J9JIM2 | GGT1 | 1565915516 | 684205807.4 | 1.194504124 | 0.042364837 | up |
| F1RGJ2 | CTNNA1 | 916859.0717 | 401237.1119 | 1.192244945 | 0.016620943 | up |
| A0A287BAY9 | ALB | 136636910.2 | 60623478.72 | 1.172398711 | 0.016735886 | up |
| P0CG68 | UBC | 171234675.3 | 76850964.18 | 1.155839614 | 0.043261751 | up |
| A0A287BKF0 | TOM1L2 | 4091947.505 | 1855663.167 | 1.140852775 | 0.019092958 | up |
| I3LD34 | MS4A14 | 8271149.438 | 3831813.448 | 1.110060509 | 0.028179239 | up |
| F1STG7 | IL1R1 | 504486.1127 | 237135.0465 | 1.08910566 | 0.034984595 | up |
| F1S7A4 | TACSTD2 | 2859959.96 | 1469912.677 | 0.960264498 | 0.018899894 | up |
| Q8SQC1 | SCARB1 | 41136195.82 | 21792274.08 | 0.91659163 | 0.032658823 | up |
| C3VPJ4 | CLDN7 | 2783357.047 | 1510990.134 | 0.881331748 | 0.036676265 | up |
| A0A287ADT7 | NA | 424922.7413 | 239428.0404 | 0.827608435 | 0.016413552 | up |
| Q28944 | CTSL | 17039052.45 | 9717549.452 | 0.810180659 | 0.038284173 | up |
| I3LRQ5 | PHACTR4 | 1186941.528 | 711859.6971 | 0.737584037 | 0.04127662 | up |
| B9TRW9 | GNG12 | 61651637.92 | 37770000.45 | 0.706898423 | 0.004408186 | up |
| B0LY42 | BSG | 1006835008 | 619803208.6 | 0.699945157 | 0.044993 | up |
| A0A287BKR0 | CTTN | 21543690.05 | 13465022.62 | 0.678048726 | 0.027782077 | up |
| Q29122 | MYO6 | 563578.7636 | 364498.0774 | 0.628706045 | 0.030025196 | up |
| A0A287B4D9 | HRAS | 49457778.33 | 32003260.79 | 0.627978527 | 0.040124984 | up |
| A0A287BKE2 | NDRG1 | 1062071.527 | 691138.3399 | 0.619834512 | 0.03203884 | up |
| A0A286ZPA9 | PSP-I | 18388442617 | 32164087580 | -0.806651465 | 0.029323987 | down |
| A0A287AYY7 | LRRC47 | 172374.4808 | 345062.2064 | -1.001310263 | 0.04080717 | down |
| F1RXD6 | CNGB3 | 918360.4062 | 2052611.421 | -1.160328189 | 0.003041862 | down |
| A0A287BKF4 | SORBS2 | 155591.5705 | 471393.4513 | -1.599167815 | 0.016480964 | down |
| F1S827 | SERBP1 | 348670.8841 | 1175826.809 | -1.753737776 | 0.020868195 | down |
| Q9TUB5 | CLCA1 | 378096.2835 | 1514420.443 | -2.001940217 | 0.018959524 | down |
| A0A287A608 | AHNAK | 473580.3902 | NA | NA | NA | NA |
| F2Z5I5 | CSNK2A1 | 632976.8201 | NA | NA | NA | NA |
| Q3HUX1 | CD36 | 5059922.095 | NA | NA | NA | NA |
| I3LAB6 | PSMA2 | 520296.8019 | NA | NA | NA | NA |
| A9X6R4 | NA | 295522.5148 | NA | NA | NA | NA |
| F1S0N2 | ACLY | 1174226.616 | NA | NA | NA | NA |
| G9F6X8 | P4HB | 269966.3867 | NA | NA | NA | NA |
| M3TYR7 | NAPRT1 | 734376.4556 | NA | NA | NA | NA |
| A0A287AQ17 | GIPC1 | 560177.8138 | NA | NA | NA | NA |
| A0A286ZY84 | TMPRSS12 | 506304.6408 | NA | NA | NA | NA |
| A0A287A5N5 | LOC100513346 | 142951.9243 | NA | NA | NA | NA |
| F1S482 | DLGAP4 | 271075.3212 | NA | NA | NA | NA |
| F1SMX5 | CAB39 | 451134.6841 | NA | NA | NA | NA |
| A0A287AB01 | TFG | 498023.4877 | NA | NA | NA | NA |
| A0A286ZJP4 | LIPI | 947195.494 | NA | NA | NA | NA |
| I3LCG1 | CC2D1B | NA | 534848.0581 | NA | NA | NA |
| A0A287BRD8 | BAG5 | NA | 72861.37157 | NA | NA | NA |
| F1RQX5 | LY6G5C | 512178.0245 | NA | NA | NA | NA |
| K9J6K2 | UTRN | NA | 1722065.371 | NA | NA | NA |
| F1SQ60 | HEG1 | 337334.4578 | NA | NA | NA | NA |
| F1SFC1 | TFPI2 | 295684.8339 | NA | NA | NA | NA |
| F1SMF4 | ITGA2 | 298135.8496 | NA | NA | NA | NA |
| I3LN42 | GC | 361967.0094 | NA | NA | NA | NA |
| F6Q5E9 | DKK3 | 100586.4175 | NA | NA | NA | NA |
| Q06AT7 | RHOF | 253596.3638 | NA | NA | NA | NA |
| I3LQQ2 | NA | 941579.6113 | NA | NA | NA | NA |
| I3L7K2 | PLCD1 | 73575.74285 | NA | NA | NA | NA |
| F1RFY5 | INCA1 | NA | 2423245.239 | NA | NA | NA |
| F1SAR7 | CLMN | 110548.9989 | NA | NA | NA | NA |
| F2Z5T7 | PPIL3 | 149965.8194 | NA | NA | NA | NA |
| A0A287AP24 | SPHK1 | 260367.5443 | NA | NA | NA | NA |
| P47788 | THOP1 | 381820.5978 | NA | NA | NA | NA |
